# p38 MAPK priming boosts VSMC proliferation and arteriogenesis by promoting PGC1α-dependent mitochondrial dynamics

**DOI:** 10.1038/s41598-022-09757-x

**Published:** 2022-04-08

**Authors:** Álvaro Sahún-Español, Cristina Clemente, Juan Ignacio Jiménez-Loygorri, Elena Sierra-Filardi, Leticia Herrera-Melle, Aurora Gómez-Durán, Guadalupe Sabio, María Monsalve, Patricia Boya, Alicia G. Arroyo

**Affiliations:** 1 Department of Vascular Pathophysiology, Centro Nacional de Investigaciones Cardiovasculares (CNIC), Madrid, Spain; 2 Department of Molecular Biomedicine, Centro de Investigaciones Biológicas Margarita Salas (CIB-CSIC), Ramiro de Maeztu 9, 28040 Madrid, Spain; 3 Department of Cellular and Molecular Biology, Centro de Investigaciones Biológicas Margarita Salas (CIB-CSIC), Madrid, Spain; 4 Department of Myocardial Pathology, Centro Nacional de Investigaciones Cardiovasculares (CNIC), Madrid, Spain; 5 Instituto de Investigaciones Biomédicas Alberto Sols (IIB), Madrid, Spain

**Keywords:** Biological models, Imaging, Microscopy, Cell growth, Cell signalling, Cellular imaging, Biological techniques, Cell biology, Physiology, Cardiovascular biology

## Abstract

Vascular smooth muscle cell (VSMC) proliferation is essential for arteriogenesis to restore blood flow after artery occlusion, but the mechanisms underlying this response remain unclear. Based on our previous findings showing increased VSMC proliferation in the neonatal aorta of mice lacking the protease MT4-MMP, we aimed at discovering new players in this process. We demonstrate that MT4-MMP absence boosted VSMC proliferation in vitro in response to PDGF-BB in a cell-autonomous manner through enhanced p38 MAPK activity. Increased phospho-p38 in basal MT4-MMP-null VSMCs augmented the rate of mitochondrial degradation by promoting mitochondrial morphological changes through the co-activator PGC1α as demonstrated in PGC1α^−/−^ VSMCs. We tested the in vivo implications of this pathway in a novel conditional mouse line for selective MT4-MMP deletion in VSMCs and in mice pre-treated with the p38 MAPK activator anisomycin. Priming of p38 MAPK activity in vivo by the absence of the protease MT4-MMP or by anisomycin treatment led to enhanced arteriogenesis and improved flow recovery after femoral artery occlusion. These findings may open new therapeutic opportunities for peripheral vascular diseases.

## Introduction

The proliferation of vascular smooth muscle cells (VSMCs) is necessary for the formation of arteries during development but also in the adult for the growth and remodeling of preformed collaterals which occurs after artery occlusion in a process called arteriogenesis^1,2^. This response is essential to restore blood flow and it is associated with a good clinical prognosis^3^. A variety of factors and pathways can promote VSMC proliferation and consequently arteriogenesis. PDGF-BB is the principal VSMC mitogen in pathophysiological contexts ranging from arterial development ^4^ to post-occlusion arteriolar remodeling^5–7^. Nitric oxide^8^, ion channels^9^ and other VSMC activators such as FGF^7^, TGF-β^10^ or Thymosin-β4 (Tβ4)^11^ also stimulate arteriogenesis by enhancing VSMC proliferation. However, the underlying signaling pathways are not fully understood.

Increased fluid shear stress after artery occlusion triggers activation of endothelial cells and subsequent recruitment of inflammatory cells^1,12^, but it is unclear how VSMCs detect and integrate these environmental signals to stimulate their proliferation. The fact that metalloproteinases (MMPs), endopeptidases responsible for the processing of extracellular matrix (ECM) components, are upregulated^13^ and capable of modulating arteriogenesis^13,14^ suggests that ECM cues may be relevant for this process^15^. Our group has discovered that GPI-anchored MT4-MMP regulates the balance between VSMC patterning and proliferation in the aorta^16^. MT4-MMP-mediated cleavage of the matricellular protein osteopontin (Opn) induces JNK signaling in VSMCs, promoting their proper positioning in the developing mouse neonatal aorta^16^. Concomitantly, MT4-MMP deficiency leads to increased VSMC proliferation in said aorta^16^.

In this work, we identify that priming of p38 MAPK by the absence of MT4-MMP or by treatment with anisomycin increases the proliferation of VSMCs in response to mitogens through PGC1α-promoted mitochondrial dynamics in vitro and it improves arteriogenesis post-artery occlusion in vivo. These findings open new avenues for VSMC-targeted preconditioning strategies in ischemic arterial disease.

## Results

### Loss of MT4-MMP increases p38 MAPK signaling in basal VSMCs in vitro, leading to their enhanced proliferation in response to PDGF-BB

**Figure 1:**
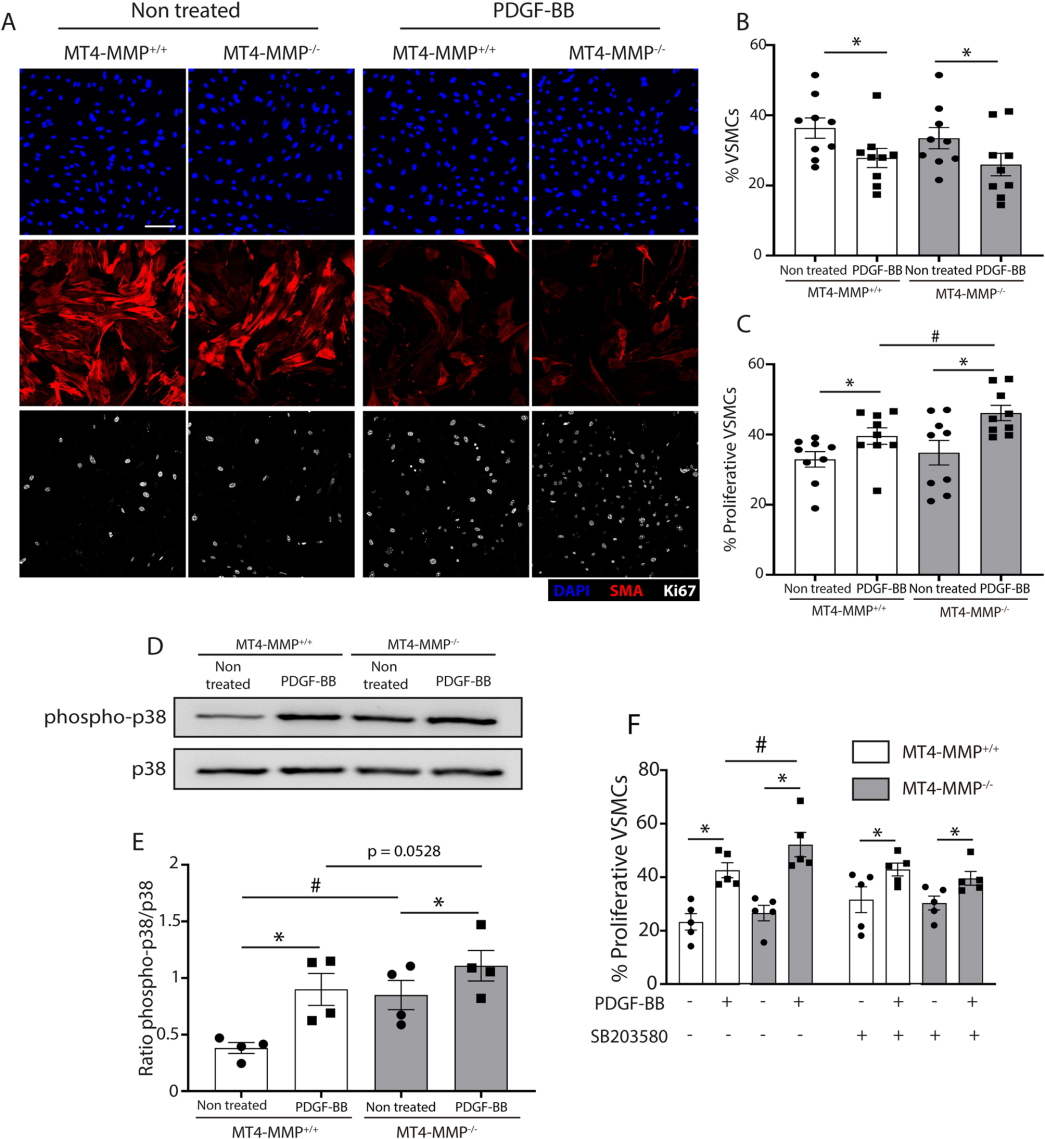
p38 MAPK signaling is increased in MT4-MMP-null VSMCs in vitro leading to boosted VSMC proliferation upon PDGF-BB treatment. (**A**) Representative confocal microscopy images showing immunostaining for SMA, Ki67 and DAPI in MT4-MMP^+/+^ and MT4-MMP^−/−^ aortic VSMCs treated or not with PDGF-BB. Scale bar: 50 µm. (**B**,**C**) Quantification of the percentage of VSMCs (% SMA^+^ in DAPI^+^ cells) and proliferative VSMCs (% Ki67^+^ in SMA^+^ cells). n = 9 VSMC cultures in 9 independent experiments. (**D**) Representative Western blot of phospho-p38 and p38 in lysates of MT4-MMP^+/+^ and MT4-MMP^−/−^ aortic VSMCs treated or not with PDGF-BB. (**E**) Quantification of phospho-p38 normalized to total p38. n = 4 VSMC cultures in 4 independent experiments. (**F**) Quantification of the percentage of proliferative VSMCs (% Ki67^+^ in SMA^+^ cells) in MT4-MMP^+/+^ and MT4-MMP^−/−^ aortic isolated VSMCs treated or not with PDGF-BB in presence or absence of p38 inhibitor (SB203580). n = 5 VSMC cultures in five independent experiments. In (**B**,**C**,**E**,**F**) data are means ± s.e.m. analysed by two-way ANOVA followed by Benjamini and Hochberg post-test; *, ^#^ p < 0.05.

We first established an in vitro model of VSMCs isolated from the aorta of young mice and stimulated them with the mitogen PDGF-BB^4^ to mimic the developmental context^17^. While PDGF-BB treatment reduced the expression of the maturation marker smooth muscle actin (SMA)^18^ in both MT4-MMP^+/+^ and MT4-MMP^−/−^ VSMCs (Fig. 1A,B), it significantly increased the proliferation of MT4-MMP^−/−^ VSMCs compared to their wild type counterparts (Fig. 1A,C). These results demonstrated the MT4-MMP cell autonomous actions on VSMC proliferation and recapitulated the increased VSMC proliferation observed in neonatal aorta, giving us the opportunity to further investigate the underlying mechanism.

In neonatal MT4-MMP^−/−^ mice, impaired cleavage of MT4-MMP substrate osteopontin (Opn) reduced the abundance of the N-terminal Opn fragment and pJNK signaling resulting in aortic wall VSMC mispositioning^16^. Decreased Opn cleavage would also lead to the accumulation of the full-length Opn protein reported to stimulate VSMC proliferation^19,20^ by its binding to αvβ3 integrin^21^ or through p38 MAPK signaling^22^. By exploring this last possibility, we found significantly higher levels of Thr180/Tyr182 phosphorylated-p38 MAPK (pp38) in cultured MT4-MMP^−/−^ VSMCs in non-stimulated (basal) conditions but not after its induction with PDGF-BB in which pp38 levels were similar in VSMCs from either genotype (Fig. 1D,E).

To determine if p38 MAPK activation in basal MT4-MMP^−/−^ VSMCs may underlie their proliferation phenotype in response to PDGF-BB, we used the α/β p38 MAPK isoform inhibitor SB203580^23^. p38 MAPK inhibition abrogated the enhanced PDGF-BB-induced proliferation in MT4-MMP^−/−^ versus WT VSMCs (Fig. 1F and Supplementary Fig. S1) but it did not prevent the induction of proliferation by PDGF-BB in either WT or MT4-MMP-null VSMCs. These data indicate that while p38 MAPK seems not essential for PDGF-BB-induced VSMC proliferation, increased pp38 MAPK in basal MT4-MMP^−/−^ VSMCs boosts the PDGF-BB-induced proliferative response.

### p38 MAPK priming promotes mitochondrial dynamics in MT4-MMP^−/−^ VSMCs

**Figure 2:**
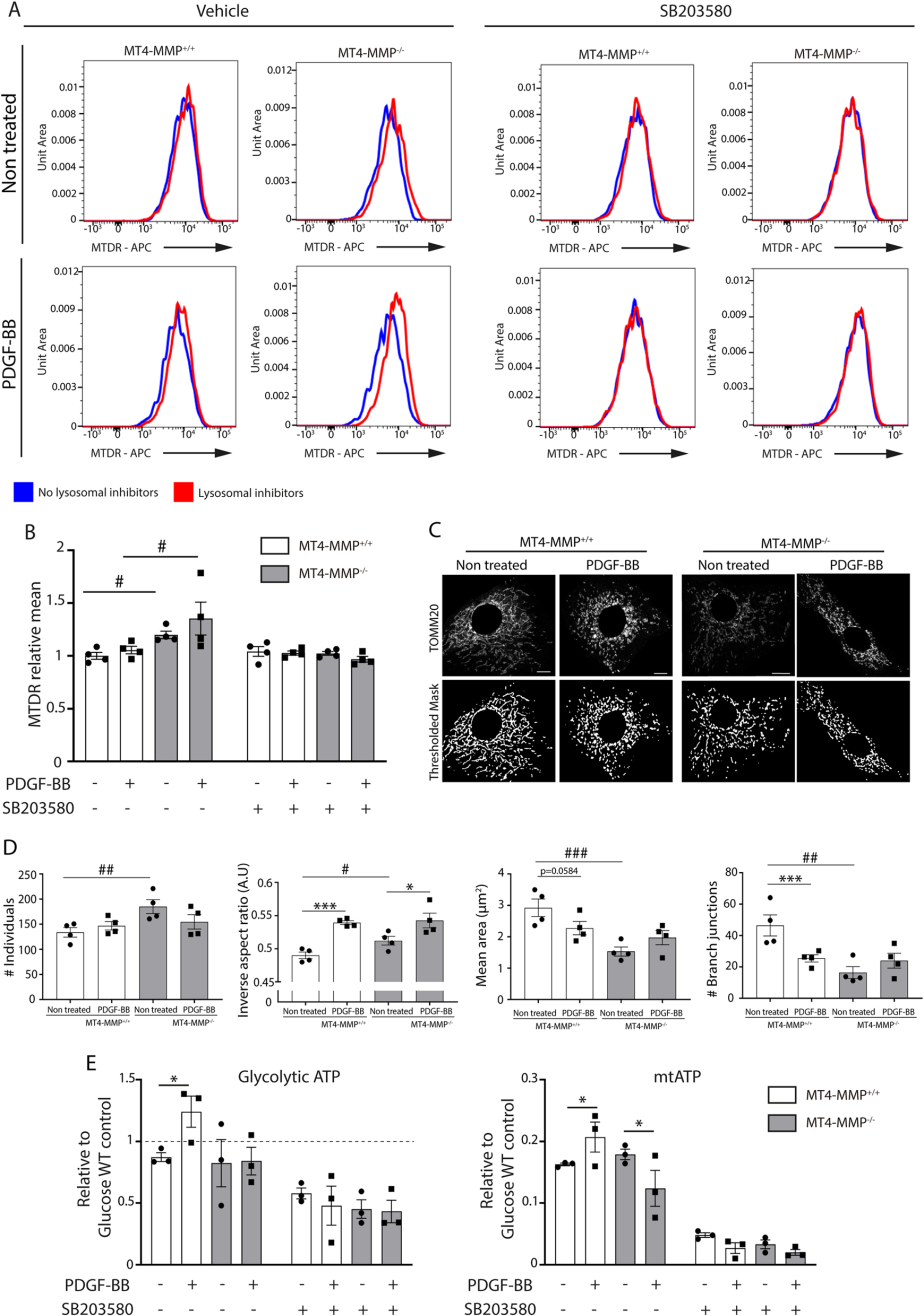
MT4-MMP deletion boosts mitochondrial degradation rate and increases mitochondrial fission in cultured VSMCs through p38 MAPK signaling. (**A**) Representative flow cytometry histogram plots of MTDR signal in MT4-MMP^+/+^ and MT4-MMP^−/−^ aortic isolated VSMCs treated or not with PDGF-BB in presence or absence of lysosome inhibitors and p38 inhibitor (SB203580). (**B**) Quantification of the mitochondrial degradation rate (MTDR MFI in the presence of lysosomal inhibitors divided by MTDR MFI in the absence of lysosomal inhibitors) normalized to MT4-MMP^+/+^ without PDGF-BB or p38 inhibitor. n = 4 VSMC cultures in 4 independent experiments. (**C**) Representative confocal microscopy images showing immunostaining for TOMM20 and its thresholded binary image of MT4-MMP^+/+^ and MT4-MMP^−/−^ VSMCs treated or not with PDGF-BB. Scale bar: 10 µm. (**D**) Quantification of number of individuals, inverse aspect ratio, mean area and number of branch junctions. n = 4 VSMC cultures in 4 independent experiments. (**E**) Quantification of relative levels of glycolytic and mitochondrial ATP in technical triplicates of MT4-MMP^+/+^ and MT4-MMP^−/−^ VSMCs treated or not with PDGF-BB and in absence or presence of SB203580. n = 3 VSMC cultures in 3 independent experiments. In (**B**,**D**,**E**) data are means ± s.e.m. analysed by two-way ANOVA with Benjamini and Hochberg post-test; *, ^#^ p < 0.05, ^##^ p < 0.01, ***, ^###^ p < 0.001.

We next explored the possible mechanism linking p38 MAPK priming in basal MT4-MMP-null VSMCs with their boosted proliferation in response to PDGF-BB. Our previous proteomics data revealed a significant decrease in the GO BP Mitochondrion in VSMC-rich aortas from MT4-MMP^−/−^ neonatal and adult mice compared to wild types^16^. Since in addition to p38 MAPK, mitochondria are relevant for VSMC proliferation^24^, we investigated these findings in more detail. As a first approach we analysed mitochondria abundance by MitoTracker Deep Red (MTDR) staining and flow cytometry in the absence or presence of lysosomal inhibitors^25^. There were significantly increased levels of MTDR signal in basal and in PDGF-BB treated MT4-MMP^−/−^ VSMCs in the presence of lysosome inhibitors (Fig. 2A,B and Supplementary Fig. S2A,B), indicating a significantly higher lysosome-degradation rate^25^ compared to wild type VSMCs. Immunofluorescence of TOMM20 and LAMP1 also showed a trend to more mitochondria inside lysosomes in MT4-MMP^−/−^ VSMCs in both basal and after PDGF-BB treatment conditions (Supplementary Fig. S2C,D), in agreement with MTDR data. Moreover, p38 MAPK inhibition with SB203580 abrogated the increased mitochondrial turnover observed by flow cytometry in MT4-MMP^−/−^ VSMCs (Fig. 2A,B).

To better understand increased mitochondrial turnover, we analysed the morphology of the mitochondrial network. Adapting an ad hoc available ImageJ plug-in^26^, we found that basal MT4-MMP^−/−^ VSMCs presented a more fragmented and globular mitochondrial network, as indicated by the significantly larger numbers and smaller size of the elements, their greater roundness and less abundance of branch junctions (Fig. 2C,D). Furthermore, PDGF-BB was able to promote mitochondrial fragmentation in WT VSMCs, but did not further change the mitochondrial network in MT4-MMP^−/−^ VSMCs (Fig. 2C,D). These results indicate that p38 MAPK priming in basal MT4-MMP^−/−^ VSMCs mainly impacts the rate of mitochondrial fragmentation and degradation. We then investigated the possible role of mitochondrial fragmentation in the enhanced VSMC proliferation observed in MT4-MMP^−/−^ VSMCs. As shown in Supplementary Fig. S3, treatment with mitochondrial division inhibitor 1 (mdivi-1)^27^ abrogated the boosted PDGF-BB-induced proliferation phenotype in MT4-MMP^−/−^ VSMCs and it also prevented the increase in proliferation normally induced by PDGF-BB in both WT, as previously reported^24^, and MT4-MMP^−/−^ VSMCs.

To gain insight into how p38-driven mitochondrial fragmentation may influence proliferation, we assessed ATP production in VSMCs in the absence or presence of 2-deoxyglucose, to estimate glycolytic and mitochondrial ATP, respectively. We confirmed that basal WT VSMCs are mostly glycolytic (> 80%) and that PDGF-BB increased glycolytic and slightly mitochondrial ATP (Fig. 2E)^28,29^. By contrast, despite no major changes in basal ATP, PDGF-BB failed to increase glycolysis and even reduced mitochondrial ATP production in MT4-MMP-deficient VSMCs (Fig. 2E). p38 MAPK inhibition decreased ATP levels in all conditions and abrogated the different behavior of MT4-MMP-null VSMCs in response to PDGF-BB (Fig. 2E). Our results indicate that p38-mediated enhanced mitochondrial fragmentation in MT4-MMP-null VSMCs impairs their production of ATP by mitochondria but also by glycolysis in response to PDGF-BB, suggesting that their increased proliferation may occur via other metabolic adaptations, such as the redirected use of glucose or its metabolites for biosynthesis of molecules^28^.

### PGC1α is required for p38 MAPK-dependent regulation of VSMC mitochondrial dynamics and increased proliferative response of VSMCs to PDGF-BB

**Figure 3:**
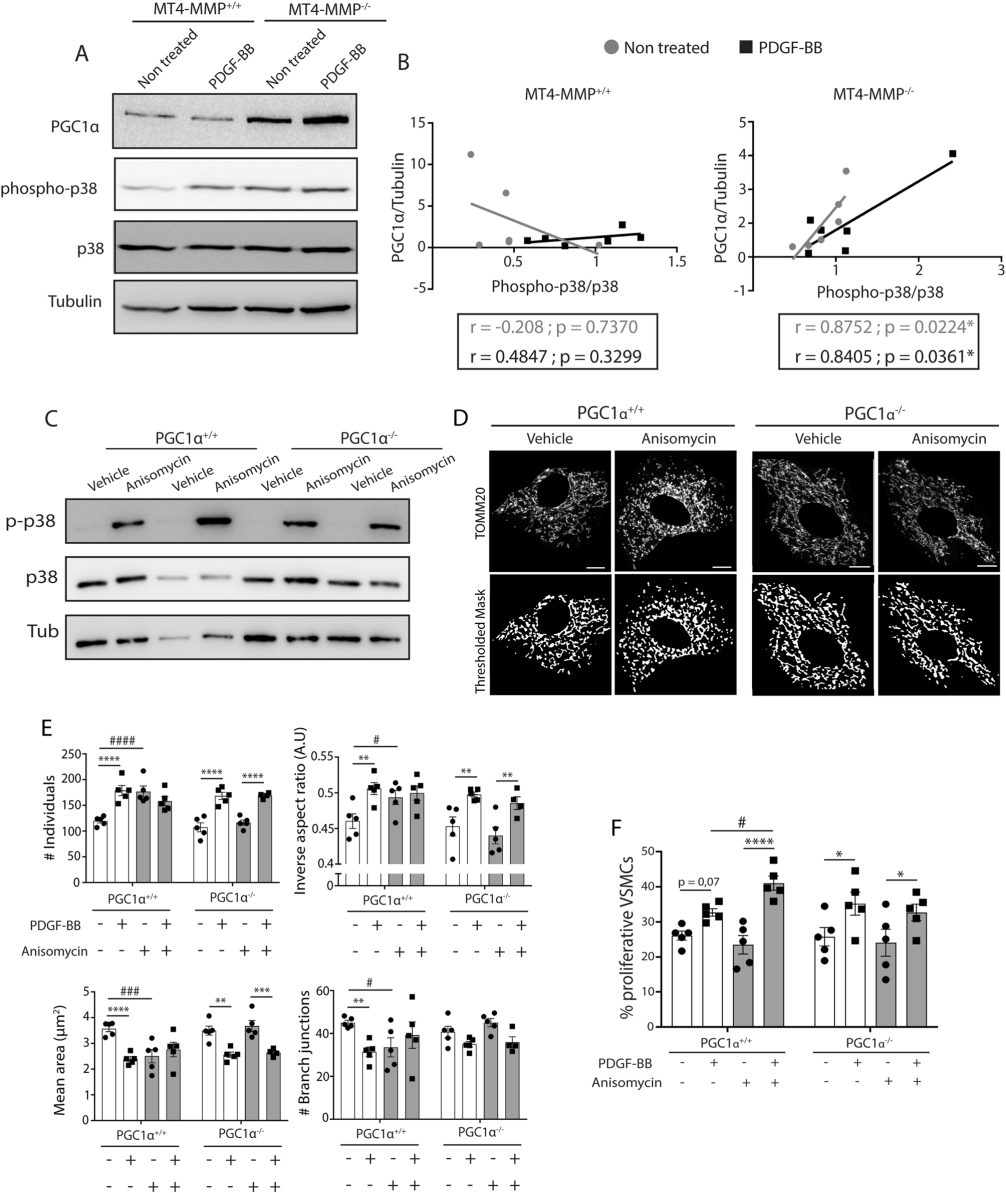
p38 MAPK priming by anisomycin treatment leads to increased mitochondrial fragmentation and PDGF-BB-induced proliferation in VSMCs in a PGC1α-dependent manner. (**A**) Representative western blot of PGC1α, phospho-p38, p38 and Tubulin in MT4-MMP^+/+^ and MT4-MMP^−/−^ VSMCs treated or not with PDGF-BB. (**B**) Correlation graphs between phospho-p38/p38 and PGC1α/Tubulin in MT4-MMP^+/+^ and MT4-MMP^−/−^ VSMCs treated or not with PDGF-BB. n = 6 VSMC cultures in 6 independent experiments. Correlation was analysed by a simple linear regression test. (**C**) Western blot of phospho-p38, p38 and Tubulin in two independent batches of PGC1α^+/+^ and PGC1α^−/−^ VSMCs pre-treated with vehicle (DMSO) or anisomycin. (**D**) Representative confocal microscopy images showing immunostaining for TOMM20 and its thresholded binary image of PGC1α^+/+^ and PGC1α^−/−^ VSMCs pre-treated or not with vehicle or anisomycin. Cells were also treated or not afterwards with PDGF-BB (images not shown). Scale bar: 10 µm. (**E**) Quantification of number of individuals, inverse aspect ratio, mean area and number of branch junctions. n = 5 VSMC cultures in 5 independent experiments. (**F**) Quantification of the percentage of proliferative VSMCs (DAPI^+^/SMA^+^/Ki67^+^) in PGC1α^+/+^ and PGC1α^−/−^ VSMCs pre-treated with vehicle (DMSO) or anisomycin and in presence or absence of PDGF-BB. n = 5 VSMC cultures in five 5 independent experiments. In (**E**,**F**) data are means ± s.e.m. analysed by two-way ANOVA with Benjamini and Hochberg post-test; *, ^#^ p < 0.05, ** p < 0.01, ***, ^###^ p < 0.001, ****, ^####^ p < 0.0001.

Next, we explored possible actors downstream of pp38 MAPK priming. Among the different substrates of p38 MAPK, peroxisome proliferator-activated receptor coactivator 1 alpha (PGC1α) is a key regulator of metabolism^30^ that signals for mitochondrial biogenesis^31,32^, and can also modulate mitochondrial dynamics^33,34^ thus contributing to overall improvement of mitochondrial quality control^35^. Given that phosphorylation of PGC1α by pp38 may stabilize the protein^36^, we performed a correlation analysis and found a significant positive correlation between the abundance of PGC1α protein and the pp38/p38 ratio in MT4-MMP^−/−^ VSMCs, basal or treated with PDGF-BB, in contrast to the absence of correlation in WT VSMCs (Fig. 3A,B).

To directly determine the involvement of PGC1α in the pp38 MAPK-induced phenotype, we treated PGC1α^−/−^ VSMCs with anisomycin (a p38 MAPK stimulator^37^) for 2 h and confirmed that it induced p38 MAPK phosphorylation (Fig. 3C) without affecting phospho-JNK levels (Supplementary Fig. S4). Anisomycin pre-treatment changed the morphology of mitochondria towards more globular and fragmented in wild-type cells but not in PGC1α^−/−^ VSMCs under basal conditions (Fig. 3D,E). Moreover, PDGF-BB induced significantly greater proliferation in wild-type cells treated with anisomycin but not in their PGC1α-deficient counterparts (Fig. 3F). These data demonstrate that PGC1α is a required actor downstream of p38 MAPK activation to regulate mitochondrial morphology changes and fragmentation in basal VSMCs and to boost their proliferation in response to PDGF-BB.

### MT4-MMP deficiency in VSMCs enhances their proliferation in remodeled arterioles and improves adductor blood flow restoration post-femoral artery occlusion

These in vitro findings led us to explore whether these signals were maintained and beneficial in pathological settings that involve VSMC proliferation such as the remodeling of pre-existent arterioles by arteriogenesis after acute arterial occlusion in vivo^1^. For that we used the femoral and epigastric artery ligation and transection procedure (see “Materials and methods”^38^) to induce collateral remodeling and arteriogenesis in the adductor muscle (Supplementary Fig. S5A,B). In addition, and since MT4-MMP is expressed by endothelial cells and monocytes and macrophages in addition to VSMCs^2,39,40^, we developed a novel conditional mouse model able to selectively delete MT4-MMP in VSMCs. An MT4-MMP floxed mouse line (herein MT4-MMP^f/f^) was generated by flanking *Mmp17* exon 2 with loxP sites (Supplementary Fig. S6). After confirming that Sm22 was specifically expressed in VSMCs of the arterioles in the superficial adductor muscle (Supplementary Fig. S7A), we crossed MT4-MMP^f/f^ mice with the Sm22-Cre transgenic mouse line^41^ to obtain a mouse line that constitutively lacks MT4-MMP in VSMCs (MT4-MMP^ΔVSMC^). We validated the selective deletion of MT4-MMP in VSMCs by comparing MT4-MMP expression in VSMCs and endothelial cells isolated from aortas of MT4-MMP^−/−^ (global deletion) and MT4-MMP^ΔVSMC^ (conditional deletion) mice (Supplementary Fig. S7B). We also confirmed that MT4-MMP^ΔVSMC^ mice displayed the same VSMC phenotype as global MT4-MMP-null mice^16^ both in the aortas from 7 day-old mice with an increase in VSMC density and in the number of mitotic VSMCs and in the aortas from adult mice with an altered pattern of VSMCs, as shown by calponin immunostaining (Supplementary Fig. S7C-G).

**Figure 4:**
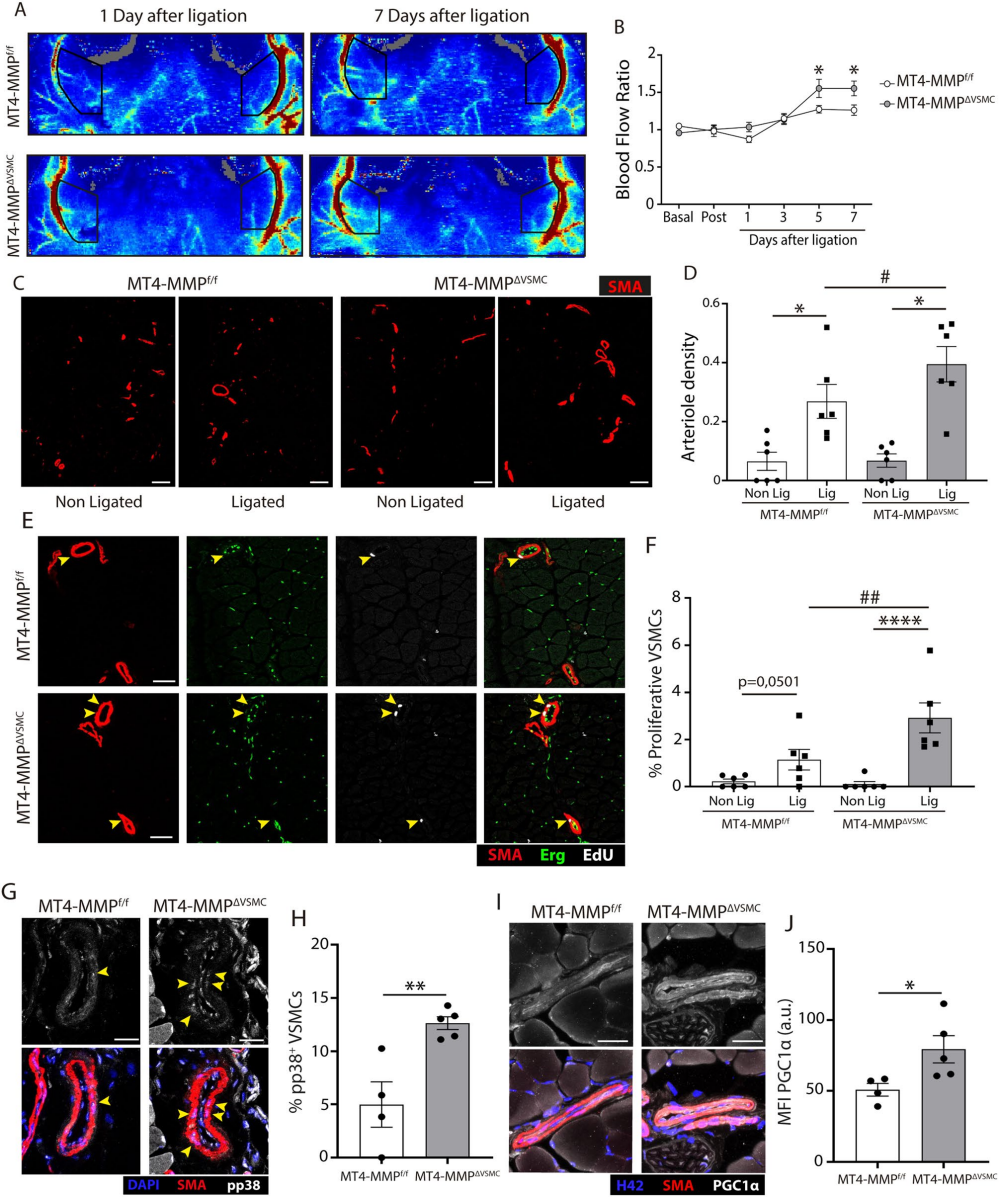
Enhanced blood flow restoration and increased VSMC proliferation and p38 activity in remodeled arterioles after femoral ligation in MT4 MMP^ΔVSMC^ mice. (**A**) Representative laser Doppler images showing Non Ligated and Ligated adductor blood perfusion 1 and 7 days after ischemia in MT4-MMP^f/f^ and MT4-MMP^ΔVSMC^ mice. (**B**) Quantification of the adductor blood flow perfusion (Non Ligated versus Ligated). n = 12 mice per genotype in 2 independent experiments. (**C**) Representative confocal microscopy images showing immunofluorescence for SMA from the superficial Non Ligated and Ligated MT4-MMP^f/f^ and MT4-MMP^ΔVSMC^ adductor muscles. Scale bar: 100 µm. (**D**) Quantification of the density of remodeled arterioles (#Arterioles > 40 µm of diameter/mm^2^ tissue) in the superficial Non Ligated and Ligated MT4-MMP^f/f^ and MT4-MMP^ΔVSMC^ adductor muscles. n = 6 mice per genotype in 2 independent experiments. (**E**) Representative confocal microscopy images showing SMA, Erg and EdU from the superficial Ligated MT4-MMP^f/f^ and MT4-MMP^ΔVSMC^ adductor muscles. Scale bar: 50 µm. (**F**) Quantification of the percentage of proliferative VSMCs (Erg^-^/SMA^+^/EdU^+^) within the remodeled arterioles. n = 6 mice per genotype in 2 independent experiments. (**G**) Representative confocal microscopy images showing immunostaining for SMA, phospho-p38 MAPK and DAPI within the remodeled arterioles. Scale bar: 25 µm. Yellow arrowheads mark VSMCs positive for phospho-p38. (**H**) Quantification of the percentage of phospho-p38^+^ VSMCs within the remodeled arterioles. n = 4 MT4-MMP^f/f^ and 5 MT4-MMP^ΔVSMC^ in two independent experiments. (**I**) Representative confocal microscopy images showing immunostaining for SMA, PGC1α and Hoechst in VSMCs within the remodeled arterioles from MT4-MMP^f/f^ and MT4-MMP^ΔVSMC^ ligated adductor muscles. Scale bar: 25 µm. (**J**) Quantification of PGC1α mean signal intensity in VSMCs within the remodeled arterioles from MT4-MMP^f/f^ and MT4-MMP^ΔVSMC^ ligated adductor muscles. In (**B**,**D**,**F**) data are means ± s.e.m. analysed by two-way ANOVA with Benjamini and Hochberg post-test. In (**H**, **J**) data are means ± s.e.m. analyzed by unpaired t-test. *, ^#^ p < 0.05, **, ^##^ p < 0.01, **** p < 0.0001.

MT4-MMP^ΔVSMC^ mice showed significant enhanced blood flow recovery at 5 and 7 days (Fig. 4A,B) and an increased number of remodeled arterioles (defined as those with a diameter larger than 40 µm^42^) in the superficial adductor muscle 7 days after surgery compared to MT4-MMP^f/f^ controls (Fig. 4C,D). Moreover, this was accompanied by a significant higher proportion of proliferative VSMCs (SMA^+^/EdU^+^) in the remodeled arterioles (Fig. 4E,F). A complementary flow cytometry analysis of the adductor muscle 7 days post-occlusion (Supplementary Fig. S8A) showed, as expected, more proliferating VSMCs in the ligated muscle of both MT4-MMP^f/f^ and MT4-MMP^ΔVSMC^ mice compared to the contralateral control muscle (Supplementary Fig. S8D,E). However, no differences between genotypes could be observed, probably related to the analysis of all arterioles and small vessels surrounded by PDGFRβ^+^ perivascular cells in the flow cytometry compared to the immunofluorescence analysis. Nevertheless, there was a significantly higher percentage of VSMCs in the ligated muscle of MT4-MMP^ΔVSMC^ mice (Supplementary Fig. S8B,C), in line with the increased remodeled arteriole density quantitated by image analysis. In this in vivo context, MT4-MMP^ΔVSMC^ mice also contained a higher percentage of positivity for pp38 and higher levels of PGC1α but no significant changes in mitolysosome abundance in VSMCs in the remodeled arterioles of the superficial adductor 7 days post-femoral artery occlusion (Fig. 4G–J; Supplementary Fig. S9A).

### p38 MAPK priming enhances arteriogenesis after femoral artery ligation

**Figure 5:**
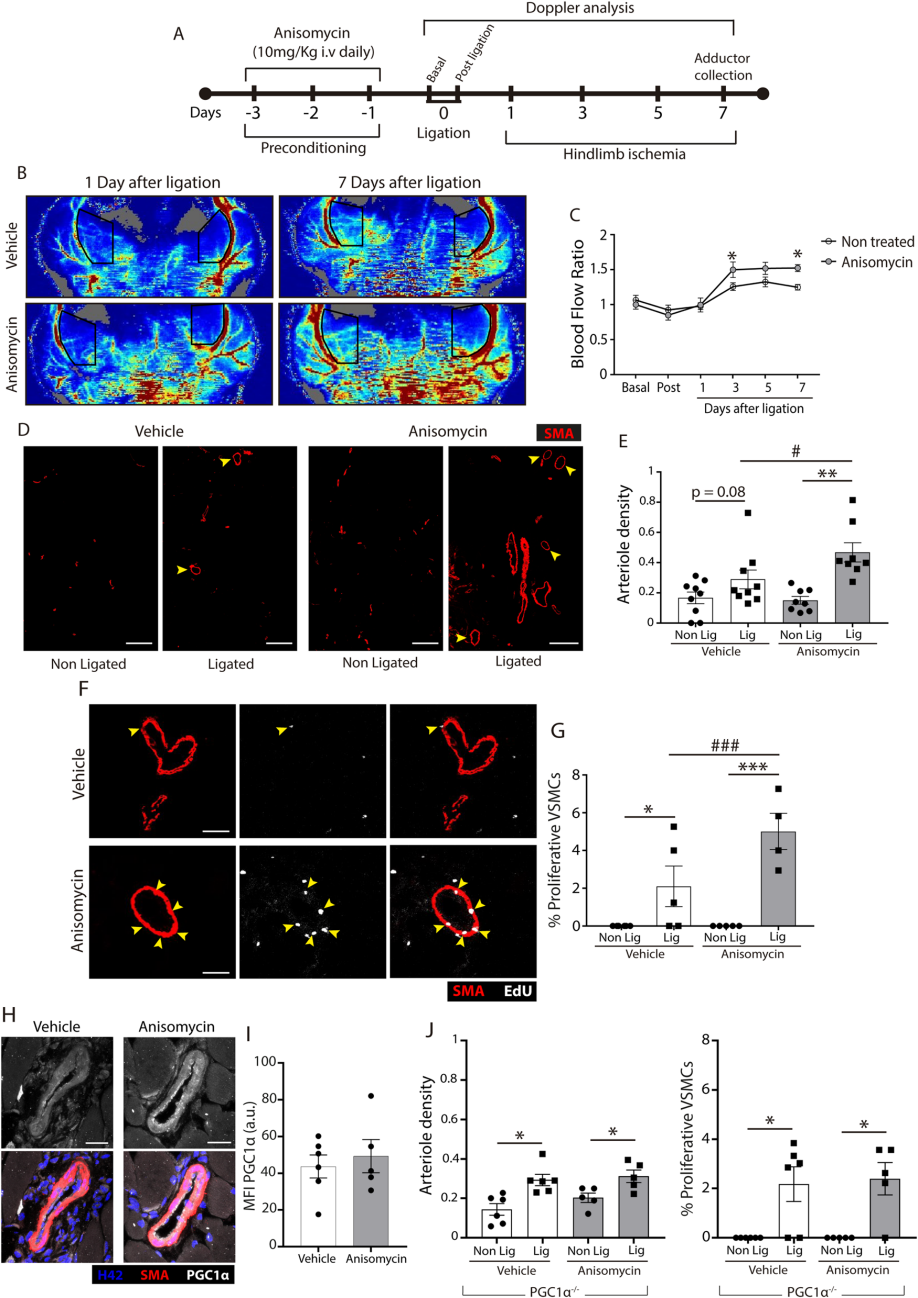
p38 MAPK priming by anisomycin pre-treatment increases blood flow restoration, arteriolar remodeling and VSMC proliferation after femoral ligation. (**A**) Scheme of the experimental design for anisomycin preconditioning in vivo and its analysis. (**B**) Representative laser Doppler images showing Non Ligated and Ligated adductor blood 1 and 7 days after ischemia in MT4-MMP^+/+^ mice pre-treated with vehicle or anisomycin. (**C**) Quantification of the adductor blood flow perfusion (Non Ligated versus Ligated ratio). n = 9 vehicle mice and 8 anisomycin mice in 3 independent experiments. (**D**) Representative confocal microscopy images showing immunofluorescence for SMA from the superficial Non Ligated and Ligated adductor muscles of MT4-MMP^+/+^ mice pre-treated or not with anisomycin. Scale bar: 200 µm. (**E**) Quantification of the density of remodeled arterioles (#Arterioles > 40 µm of diameter/mm^2^ tissue) within the superficial Non Ligated and Ligated adductor muscles of wild-type mice pre-treated or not with anisomycin. n = 9 vehicle mice and 8 anisomycin mice in 3 independent experiments. (**F**) Immunofluorescence of SMA and EdU from the superficial Ligated adductor muscle of wild-type mice pre-treated or not with anisomycin. Scale bar: 50 µm. (**G**) Quantification of the percentage of proliferative VSMCs (SMA^+^/EdU^+^) within the remodeled arterioles. n = 6 vehicle mice and 5 anisomycin mice in 3 independent experiments. (**H**) Representative confocal microscopy images showing immunostaining for SMA, PGC1α and Hoechst in VSMCs within the remodeled arterioles of the ligated superficial adductors from vehicle- or anisomycin-treated mice. Scale bar: 25 µm. (**I**) Quantification of PGC1α mean signal intensity in VSMCs within the remodeled arterioles from vehicle- or anisomycin-treated mice. (**J**) Quantification of the density of remodeled arterioles (#Arterioles > 40 µm of diameter/mm^2^ tissue) (left) and the percentage of proliferative VSMCs (SMA^+^/EdU^+^) (right) within the remodeled arterioles of PGC1α-null mice pre-treated or not with anisomycin 7 days post-ligation. n = 6 vehicle mice and 5 anisomycin mice in one experiment. In (**C**,**E**,**G**,**J**) data are means ± s.e.m. analyzed by two-way ANOVA with Benjamini and Hochberg post-test. In (I) data are means ± s.e.m. analyzed by unpaired t-test.; *, ^#^ p < 0.05, **, ^##^ p < 0.01, ***, ^###^ p < 0.001, **** p < 0.0001.

Finally, we directly tested whether p38 MAPK activation per se may also enhance VSMC proliferation and arteriogenesis in vivo regardless of MT4-MMP expression. Based on our findings in the in vitro VSMC culture (Fig. 3C), we designed a p38 MAPK priming intervention consisting of pre-treating mice with 10 mg/kg anisomycin once a day intravenously for 3 days before performing femoral artery ligation for a further 7 days (Fig. 5A). Doppler imaging showed that 7 days after femoral artery ligation there was a significantly increased blood flow restoration in anisomycin-treated mice compared to those treated with vehicle (Fig. 5B,C). This improved blood flow recovery was accompanied by a significant increased number of remodeled arterioles (Fig. 5D,E) and a higher proportion of proliferating VSMC in the adductor muscles of anisomycin-treated mice after surgery (Fig. 5F,G). Although we were unable to detect differences in PGC1α levels (Fig. 5H,I), we captured a trend toward greater abundance of mitolysosomes in VSMCs from remodeled arterioles of anisomycin-treated mice in comparison with controls 7 days after femoral artery ligation (Supplementary Fig. S9B). Notably, anisomycin pre-treatment failed to increase the number of remodeled arterioles and proliferating VSMCs in PGC1α-null mice (Fig. 5J) in contrast to wild-types (Fig. 5D–G), demonstrating the in vivo PGC1α requirement for anisomycin-induced VSMC proliferative response.

## Discussion

**Figure 6:**
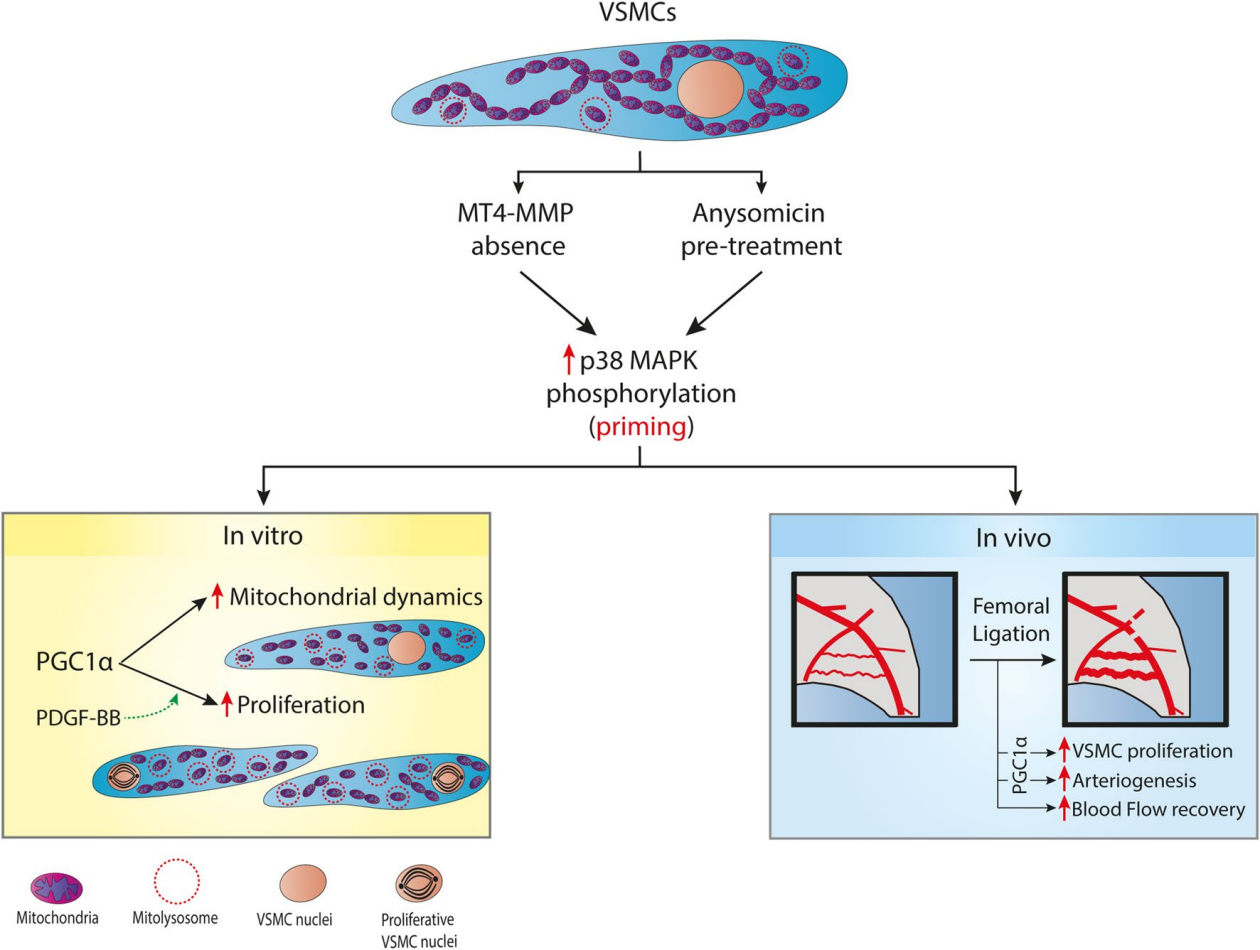
Graphical abstract: An increase in p38 MAPK phosphorylation in VSMCs (caused by external preconditioning with anisomycin or by knockdown of MT4-MMP) leads to a basal primed state in which mitochondrial dynamics is enhanced via PGC1α. This cellular context allows for a better proliferative response of VSMCs to in vitro treatment with PDGF-BB. Furthermore, in vivo, after femoral artery ligation, such VSMC priming allows for better (PGC1α-dependent) VSMC proliferation and arteriogenesis, and thus more efficient recovery of blood flow.

In this work we identified the p38 MAPK-PGC1α-mitochondrial adaptive response axis as a novel molecular pathway relevant to VSMC proliferation and arteriogenesis after femoral artery occlusion in vivo (Fig. 6).

Our group previously observed that MT4-MMP expression in the developing aortic vessel wall balanced signals for patterning versus proliferation in the VSMCs^16^. In particular, MT4-MMP Opn processing activates JNK for VSMC patterning in the embryonic and neonatal aortic vessel wall^16^. We now show that, in the absence of MT4-MMP, the expected accumulation of unprocessed Opn would increase p38 MAPK phosphorylation in MT4-MMP^−/−^ VSMCs in vitro, consistent with previous reports on the effects of Opn in VSMCs^43^ and other contexts^44,45^. Interestingly, Opn has been shown to induce VSMC apoptosis unless there is hypoxia or starvation^46^, the latter being present in our in vitro VSMC culture. Furthermore, we observed that increased p38 MAPK activity and mitochondrial fragmentation does not enhance the proliferation of MT4-MMP^−/−^ VSMCs unless PDGF-BB is added, in agreement with this limited ability of p38 MAPK to modulate Opn-induced proliferation^43^ except under hypoxia^22^, other conditions of cellular stress^47^, or in complex in vivo scenarios^48,49^. Regarding the possible contribution of other substrates and/or signals to p38 MAPK activity in MT4-MMP-null VSMCs, HB-EGF and EGFR can regulate p38 MAPK and cell proliferation^50,51^ and both are regulated by cleavage or signaling by MT4-MMP^52,53^. However, in that case, the absence of MT4-MMP would dampen rather than enhance p38 MAPK activity. In addition, although other MMPs (MMP3, 7, 9 and 12) can cleave Opn^54^, they generate different Opn fragments and their levels are not affected in MT4-MMP-null VSMCs^16^, so their contribution to the phenotype observed seems unlikely.

Our results recall adaptive mitochondrial responses, which are also capable of triggering proliferation under stress conditions such as hypoxia and/or starvation^55^. In addition, and in accordance with the changes observed in mitochondrial fragmentation, morphology and turnover, mitochondrial dynamics have been found associated with the proliferation of VSMCs^24^. Indeed, mitochondrial fragmentation (fission) is necessary for VSMCs proliferation^56^ while mitochondrial fusion leads to anti-proliferative responses^57^. In the pp38 priming context, mitochondrial fragmentation recalls mitohormetic responses^58^ in which cells are prepared to cope better with a subsequent stress or signal as the mitogen PDGF-BB.

By using PGC1α-deficient VSMCs, we have demonstrated that PGC1α is a required actor downstream of p38 MAPK activation to regulate mitochondrial morphology changes and fragmentation in non-treated VSMCs and to boost their proliferation in response to PDGF-BB. In the same line, PGC1α has recently been shown to be beneficial in acute renal stress by inducing an adaptive mitohormetic-like response, thus maintaining mitochondrial homeostasis^59^. PGC1α also participates in cell metabolism^60^, which may contribute to increase the proliferative capacity in VSMCs. In fact, VSMC proliferation associated with mitochondrial fission was reported to occur concomitantly with a change in metabolism^24^. Our ATP data show that enhanced mitochondrial fragmentation in MT4-MMP-null VMSCs results in decreased mitochondrial ATP in response to PDGF-BB and, since glycolysis also appears to be impaired, it may favor other metabolic changes leading, for example, to a more prominent redirected use of glucose or its metabolites for the biosynthesis of molecules for proliferation^28^. PGC1α will be an essential coordinator of mitochondrial dynamics, metabolic adaptations and proliferation in VSMCs, perhaps, at least in part, through its transcriptional target CPT1^29,61^.

The in vitro findings led us to explore whether these signals were maintained and beneficial in pathological settings that involve VSMC proliferation such as the remodeling of pre-existent arterioles by arteriogenesis after acute arterial occlusion^1^. Our previous observations together with this study lead us to speculate that MT4-MMP may function as a molecular switch, translating signals from the matricellular environment to VSMCs and promoting their differentiation or proliferation depending on the context. On the one hand, acute arterial occlusion will induce mechanical stress in the vessel wall, which will stimulate Opn production by VSMCs^62^ (Opn is hardly expressed in the quiescent arterial vessel wall^63^). On the other hand, the recruited macrophages will produce PDGF-BB^64^, which will probably decrease endogenous MT4-MMP expression. The sequential effect of mechanical stretching and PDGF-BB will accumulate unprocessed Opn in VSMCs (particularly if MT4-MMP is removed) and likely induce p38 MAPK phosphorylation, since pp38 MAPK in VSMCs was barely detected in vivo under homeostatic conditions, further supporting the need for additional signals to activate this pathway (stretching or mild hypoxia)^62^. Along these lines, Opn is increased in peripheral artery disease^65^ and genetic overexpression of Opn seems to enhance arteriogenesis^66^ while its deletion impairs blood flow recovery^67^. These data are consistent with numerous reports showing that increased VSMC proliferation makes arteriogenesis more efficient in the HLI model^8,9,68^ and discover MT4-MMP as a VSMC quiescence driver. In addition, the generation of a new conditional mouse line has also allowed us to demonstrate the cell autonomous role of MT4-MMP in VSMC proliferation and arteriogenesis in vivo. Although Sm22 expression has been reported in peritoneal macrophages and blood neutrophils and monocytes^69^, cell lineages with potential beneficial actions in arteriogenesis^12,70^, we did observe the proliferative phenotype in MT4-MMP^−/−^ VSMCs cultured in vitro, arguing in favour of a cell autonomous action.

The fact that MT4-MMP is important both for arterial vasculature development^16^ and for arteriogenesis after occlusion supports the hypothesis about the recapitulation of the developmental mechanisms in adult vascular disease^71^. In fact, transcription factors such as Carp or molecules such as Cx37 influence both arterial morphogenesis^72,73^ and adult arteriogenesis^74,75^.

Our data suggest that an enhanced p38 MAPK phosphorylation establishes a primed status in VSMCs capable of mounting a more robust proliferative response to mitogenic factors under stresses such as starvation or hypoxia. Furthermore, our data of increased PGC1α expression and mitochondrial dynamics in VSMCs in ligated adductors of MT4-MMP^ΔVSMC^ and anisomycin-treated mice along with the lack of enhanced arteriogenesis in PGC1α-null mice after arterial ligation demonstrate that the newly identified axis pp38/PGC1α/mitochondrial dynamics/VSMC proliferation also works in vivo.

Hence, priming or preconditioning of this pathway due to the absence of the protease MT4-MMP or by prior treatment with the p38 MAPK activator anisomycin leads to greater PDGF-BB-induced VSMC proliferation in vitro and to better arteriogenesis and flow recovery after femoral arterial occlusion in vivo (Fig. 6).

This study, together with our previous reports on predisposition to aortic aneurysms^16^ and acceleration of atherosclerosis^40^ in mice lacking MT4-MMP, suggests a possible Janus effect^76^ to consider when proposing MT4-MMP-based therapeutic approaches for cardiovascular diseases. Interestingly, p38 MAPK has been involved in beneficial xenon^77^ and adenosine-induced^78^ preconditioning as well as in the so-called ischemic preconditioning^79^ to protect the heart and other organs, but little research has been conducted on its effects after peripheral artery occlusion. Our data demonstrate that anisomycin-induced activation of p38 MAPK also serves as a preconditioning strategy in VSMCs in the arteries of the extremities. Although in cardiac ischemia/reperfusion injury, p38 MAPK activation is beneficial as preconditioning strategy but deleterious after the event^80^, it could still be of interest to test the therapeutic effect of anisomycin after peripheral arterial occlusion given the possible contribution of context and/or tissue-dependent factors. For all the above, priming, non-systemic and/or short-term treatments based on the inhibition of MT4-MMP or the activation of p38 MAPK could be suggested due to its beneficial effects on the recovery of blood flow in peripheral ischemic vascular diseases.

## Materials and methods

### Mice

MT4-MMP^lacZ/lacZ^ mice (herein MT4-MMP^−/−^) were generated by Dr. Motoharu Seiki's laboratory^39^. MT4-MMP^flox/flox^ (herein MT4-MMP^f/f^) was generated in our group by flanking *Mmp17* exon 2 with loxP sites. MT4-MMP^ΔVSMC^ was obtained by crossing MT4-MMP^f/f^ with an *SM22α-Cre* (B6.Cg-Tg(Tagln-cre)1Her/J) mice^41^ (The Jackson Laboratory, Bar Harbor, ME USA) kindly provided from V. Andrés' laboratory (CNIC, Madrid). Pgc1α-deficient mice (herein PGC1α^−/−^) were part of a colony established in the IIB animal facility and originally derived from mice provided by Dr. Bruce Spiegelman (DFCI, Boston, USA)^81^. All mice were maintained on the C57BL/6 background and genotyped by PCR of tail samples using specific primers (Supplementary Table S1).

Mice were housed in the Centro Nacional de Investigaciones Cardiovasculares Carlos III (CNIC) Animal Facility under pathogen-free conditions and according to institutional guidelines. Experiments were performed in accordance with Spanish legislation on animal protection (2010/63EU) and animal procedures were approved by the Committee on the Ethics of Animal Experiments of the CNIC (procedure number: CNIC-01/13) and by the corresponding legal authority of the local government ‘Comunidad Autónoma’ of Madrid (permit number: PROEX 34/13). All methods are reported in accordance with ARRIVE guidelines. No statistical methods were used to pre-estimate the animal sample size and mice were randomly allocated, without exclusions, to experimental groups after pertinent age and sex considerations (referred below in each case). For the experiments of anisomycin treatment of VSMCs or mice, the investigator analyzing the data was blinded to group allocation.

### Isolation and culture of VSMCs

Four to five young mice (3- to 4-wk-old) per genotype were used to obtain VSMCs. Mice were sacrificed by CO_2_ and aorta was dissected and cleaned from fat while maintained in cold collection medium (10% FBS, 2 mM L-glutamine, 50 UI/ml penicillin, 50 μg/ml streptomycin and 25 mM HEPES in DMEM). Cleaned aortas were then incubated at 37 °C for 5 min in 200 µl of collagenase type I (LS004194, Worthington) at 3.33 mg/ml diluted in DMEM. Then, adventitia was removed and aortas were cut into small pieces (1–2 mm) that were incubated for 45 min at 37 °C in 100 µl of type I collagenase 6 mg/ml and elastase (LS002290, Worthington) at 2.5 mg/ml diluted in DMEM. Finally, cells were disaggregated and seeded. After 3–4 days, co-cultures of mouse aortic endothelial cells (MAECs) and VSMCs were washed once in PBS and then were incubated 30 min at 4 °C with Rat anti-ICAM-2 (553325, BD Biosciences) diluted 1:500 in PBS. Next, cells were washed twice and sheep anti-rat IgG magnetic beads (Dynabeads™) (11035, ThermoFisher) diluted 1:250 were added to the cells for another 30 min at 4 °C. Finally, cells were trypsinized and collected in a falcon tube inside a magnet. Supernatant (VSMCs) was placed in gelatin pre-coated plates in 10% FBS, 2 mM L-glutamine, 50 UI/ml penicillin, 50 μg/ml streptomycin and 25 mM HEPES in DMEM. VSMCs were always used at early passage 2 to prevent their dedifferentiation and reduce inter-experiment variability.

### VSMC treatment

For western blot or cytometry, VSMCs cells were seeded at 85,000 cells/well in 12-well-plates. For immunofluorescence, VSMCs cells were seeded at 4.000 cells/well in Ibidi chambers (81506, Ibidi). In both cases, when 60% confluent, starvation medium (2 mM L-glutamine, 50 UI/ml penicillin, 50 μg/ml streptomycin and 25 mM HEPES in DMEM) was added for 24 h. Next day, starvation medium was substituted by new starvation medium with or without 20 ng/ml of PDGF-BB (315–18, Preprotech) for another 24 h of treatment. For p38 inhibition, SB203580 diluted in DMSO was added at 15 µM 1 h before PDGF-BB treatment, and was maintained during the whole treatment. For mitochondria division inhibition, mdivi-1 (M0199, Sigma-Aldrich) diluted in DMSO was added at 10 µM 1 h before PDGF-BB treatment, and was maintained during the whole treatment. For p38 activation, anisomycin diluted in DMSO was added at 150 ng/ml 2 h before PDGF-BB treatment, and was removed when adding PDGF-BB.

### VSMC immunofluorescence, image acquisition and analysis

Cells were fixed in 4% PFA for 10 min RT and washed twice in PBS. Next, cells were permeabilized for 15 min at RT with 0.2% Triton X-100 in PBS, followed by a 1 h RT blocking in 5% goat serum and 5% BSA in PBS. Then primary antibodies (Supplementary Table S2) were incubated O/N at 4 °C in 2.5% goat serum and 2.5% BSA in PBS. The next day, sections were washed at RT for 10 min 3 times in PBS and secondary antibodies (Supplementary Table S2) and DAPI (1/5000) for nuclear staining were incubated for 1 h at RT in 2.5% goat serum and 2.5% BSA in PBS. Finally, cells were washed 4 times 10 min. For proliferation, cells were imaged with confocal Nikon A1R, acquiring a complete 5-z-stack tile scan of the ibidi well every 1 µm using a 10 × objective. Fiji/Image J Software was used to obtain the total number of cells by quantifying the number of DAPI^+^ nuclei. DAPI^+^/SMA^+^ were considered as VSMCs and DAPI^+^/SMA^+^/Ki67^+^ as proliferative VSMCs. The percentage of VSMCs was calculated as the number of VSMCs (SMA^+^) relative to the total number of cells (DAPI^+^), and the percentage of proliferative VSMC was calculated as the Ki67^+^/SMA^+^ number (proliferative VSMC) versus the number of VSMC (SMA^+^). For mitochondrial analysis, cells were fixed for 30 min with 4% PFA at RT and permeabilized using 0.1% SDS in PBS for 30 min at RT. Then, cells were incubated with primary antibodies (Supplementary Table S2) diluted in 3 mg/mL BSA, 100 mM Glycine, 025% Triton X-100 in PBS for 1 h at RT. Cells were then incubated with corresponding secondary antibodies (Supplementary Table S2) diluted in the same buffer for 1 h at RT and counterstained with DAPI. Three washes with PBS were performed after every step. Cells were imaged using a 63 × oil immersion objective in a TCS SP5 Confocal System (Leica). At least three images per sample were acquired. For co-localization analysis, z-stacks (z-step: 0.5 µm) were processed using JaCoP^82^ with a standard manually-adjusted threshold for the red (C1-LAMP1) and green (C2-TOMM20) channels. Mitochondrial and lysosomal mass values were obtained by quantifying the TOMM20- or LAMP1-positive area per cell, respectively, using Fiji/ImageJ. For mitochondrial net study nine cells per well and condition were analyzed with the Mitochondrial Analyser plug-in^26^.

### VSMC protein extraction and western blot

VSMCs were lysed on Ripa buffer (150 mM NaCl, 0.1% SDS, 1% NP-40, 50 mM Tris–HCl pH 8, 0.5% Sodium Deoxycholate, 5 mM EDTA) supplemented with protease (11-836-153-001, Roche) and phosphatase (04-906-845-001, Roche) inhibitors. The lysate was collected, incubated for 45 min on ice and next centrifuged at 13,000 rpm for 15 min at 4 °C. Supernatant (cell lysate) was collected in new tubes and quantified using Pierce BCA Protein Assay Kit (23227, ThermoFisher) and Microplate Manager software (version 5.2.1, Bio-Rad). Then, 25 µg of protein were reduced with 5% β-Mercaptoethanol in Laemmli buffer and boiled at 100 ºC for 5 min. Samples were run on 8% SDS–polyacrylamide gel for 1.5 h in running buffer (25 mM Tris–HCl pH 8.3, 192 mM glycine, 0.1% SDS) and then transferred for 1.5 h under 400 mA constant into 0.45 µm nitrocellulose membrane (1620115, Bio-Rad) in transfer buffer (25 mM Tris–HCl pH 8.3, 192 mM glycine, 20% Methanol). In most experiments after blocking in 5% BSA for 1 h at RT, membranes were cut prior to incubation of the fragments O/N in 2.5% BSA with the corresponding primary antibodies (Supplementary Table 4). The next day, membranes were washed 3 times 5 min in TBST and incubated 1 h RT in 2.5% BSA with 1:5000 goat anti-rabbit HRP (111-035-003, Jackson). Afterwards, membranes were washed four times 10 min in TBST and signal was developed with Luminata Immobilon Classico (WBLUC0100, Merk) in a chemiluminescence imager (LAS-4000, Life Sciences). For total p38 or JNK assessment, membranes were stripped for 30 min at 55 °C in 50 mL of stripping buffer (2% SDS, 62.5 mM Tris–HCl pH 6.8, 100 mM β-mercaptoethanol). After that, membranes were washed 3 times 15 min in TBST being afterwards blocked as previously described. Protocol proceeded equal but incubating with rabbit anti-p38 (SC535, Santa Cruz) or rabbit anti-JNK (9252, Cell Signaling) diluted 1:1000. Western Blot images were processed and quantified with Fiji/ImageJ software.

### ATP production

ATP levels were measured using the CellTiter-Glo Luminescent Cell Viability Assay (G7570, Promega) according to previously established methods^83^. Briefly, 10.000 VSMCs/well were seeded on a p96 plate and when 60% confluence was reached, cells were changed to starvation medium (0% FBS) for 24 h. The day after, in fresh starvation medium, the p38 inhibitor SB203580 was added at 15 µM 1 h before PDGF-BB treatment (20 ng/ml), and both were kept for 24 h. After that time, cells were lysed, and lysates were incubated with luciferin and luciferase reagents in order to measure ATP production using a Thermo Scientific Varioskan LUX multimode microplate reader (Thermo Fisher Scientific). Results were normalized to relative measurements of the protein quantity in a parallel plate.

### Hindlimb ischemia

Equal numbers of 12- to 15-wk-old male and female mice were used in all groups. When required, 10 mg/kg of anisomycin was intravenously injected every day during three days before the surgery. Then, mice were depilated and anesthetized under 2% isoflurane (50019100, Zoetis) at an oxygen flow rate of 2 L/min for 5 min. Surgery was performed over a heating pad at 37 °C under continuous anesthesia, following considerations of already described procedures^84^. Briefly, the femoral artery was separated from the vein and nerve, ligated distally from the proximal caudal femoral artery and proximally to the popliteal artery and transected in between. Additionally, superficial epigastric artery was also ligated and transected^38^.

### Doppler analysis

Doppler analyses were performed just before (basal) and after (post) HLI surgery and then 1, 3, 5 and 7 days after ischemia. For Doppler measurements, mice were anesthetized under 2% isoflurane at an oxygen flow rate of 2 L/min for 5 min. Then mice were placed over a heating pad at 37 ºC in supine position. Measures were performed with the infrared laser of a Doppler imager (moorLDI2-HIR, Moor LDS) and images were analyzed with MoorLDI Laser Doppler Imager Review V6.0 (MoorLDI). The regions of interest (ROIs) in the adductor area for collateral blood flow measurements were drawn slightly modified from previous studies^85^. The left boundary was drawn just outside the margin of the saphenous artery between the lateral circumflex femoral artery and the bifurcation of the tibial artery. The bottom boundary is a line perpendicularly extended from the curved line to the medial part of the body. The right boundary is a vertical line linking the bottom boundary and the zenith of the adductor curvature. Finally, the top boundary links the top edges of the left and right boundaries. The total blood flow in the region was measured and then normalized by the area of the ROI. For each mouse, blood flow of adductor in the ligated leg was normalized by the blood flow of the adductor in the non-ligated leg.

### Adductor collection and processing for immunohistochemistry and immunofluorescence

For adductor collection mice were sacrificed by means of CO_2_ at the study end-point (7 days after HLI surgery). For immunofluorescence only, mice were injected intraperitoneal with 40 mg/kg body weight of EdU (A10044, ThermoFisher) 3 h previously to their sacrifice. After sacrifice, heart perfusion was carried out with 10 ml of 4% PFA and superficial adductor muscles of ligated and non-ligated hindlimbs were dissected. Adductor muscles were post-fixed in 0.4% PFA at 4 °C, O/N. Next day, adductor muscles were embedded in 30% sucrose in PBS for 24 h at 4 °C and frozen in OCT (4583, Tissue-Tek) at − 80 °C.

### Tissue immunohistochemistry and immunofluorescence

For adductor immunohistochemistry, 5 µm sections were cut transversally to GCs. Arteriole staining was performed automatically (Autostainer Plus, Dako/Agilent) under buffer citrate pH 6 antigen retrieval and using a Rabbit anti-SMA primary antibody (RB-9010-PO, ThermoFisher) at 1/300. SMA was developed using an HRP anti-Rabbit (K400311, Dako). For adductor immunofluorescence, 10 µm sections were cut transversally to the GCs. Sections were blocked/permeabilized for 1 h at RT with blocking buffer (0.2% Triton X-100, 5% goat/donkey serum and 5% BSA in PBS) and primary antibodies (Supplementary Table S3) were incubated O/N at 4 °C in incubation buffer (0.2% Triton X-100, 2.5% goat/donkey serum and 2.5% BSA in PBS). Next day, sections were washed at RT for 10 min 3 times with washing buffer (0.1% Triton X-100 in PBS) and secondary antibodies (Supplementary Table S3) and DAPI (1/5000) for nuclear staining were incubated for 1.5 h at RT in incubation buffer. When required, right before mounting, EdU mix was prepared following Click-iT EdU Cell Proliferation Kit instructions (C10340, ThermoFisher) and added over the sections for 30 min at RT. Sections were washed 15 min twice in PBS at RT before Fluoromount-G (0100-01, Southern Biotech) mounting.

### Tissue image acquisition and analysis

For immunohistochemistry, whole slide images were acquired with a digital slide scanner (Nanozoomer-RS C110730, Hamamatsu) and then visualized using NDP.view2 software (Hamamatsu Photonics). For immunofluorescence, a confocal microscope (Nikon A1R, Nikon) was used to acquire a 20 × 5-z-stack tile-scan of the whole adductor muscle section every 1.5 µm. Fiji/Image J software was used to manually select superficial adductor muscle area, where the same software automatically selected and counted the remodeled arterioles (> 40 µm diameter)^42^ in both immunohistochemistry and immunofluorescence samples. Total number of VSMCs (DAPI^+^/SMA^+^) and proliferative VSMCs (DAPI^+^/SMA^+^/EdU^+^) were counted within the remodeled arterioles. For analysis of PGC1α in arterioles, a confocal microscope (Leica SP8) was used to acquire 63 × images of individual arterioles along a stack of 5z every 1 µm. The mean fluorescence intensity (MFI) of PGC1α was calculated for the brightest section of the z-stack to avoid the high background obtained when creating maximal projections. To analyze mitochondria in arterioles, a confocal microscope (Leica SP8) was used to acquire 63 x—2 × zoom images of individual arterioles in a stack of 5z every 0.5 µm. TOMM20^+^/LAMP1^+^ double positive events were considered as mitolysosomes and their number was normalized by VSMC area (SMA^+^ area) to obtain mitolysosome density.

### Flow cytometry in the adductor muscle

Mice were sacrificed 7 days after HLI surgery by means of CO2 and perfused with 10 ml of cold PBS. Surface adductors were dissected as previously described^86^ and collected in cold DMEM. Next, adductor muscles were diced and digested in 0.1% collagenase IV (C5138, Sigma) for 45 min at 37 °C. Tissue was gently disaggregated and filtered through a nylon mesh of 70 μm with FACS buffer (5% FBS, 5 mM EDTA in PBS). Then, samples were centrifuged at 1250 rpm for 5 min at 4 °C, supernatant was discarded, and pellet was resuspended in 1 ml of ACK buffer for 10 min at RT. Next, cells were washed centrifuging at 1250 rpm for 5 min at 4 °C in 10 ml of FACS buffer. The supernatant was removed and cells were Fc-blocked for 30 min with 100 μl of FACS buffer with a Rat anti-mouse CD16/CD32 antibody (553142, BD Pharmingen) diluted 1:100. A fixable viability dye (L34959, ThermoFisher) diluted 1:1000 was added for viability assessing. After a FACS washing step, 1:50 Rat anti-PDGFRβ-PE (136005, BioLegend) was added for 45 min at 4 °C in FACS buffer. Next, cells were fixed/permeabilized following Foxp3/Transcription Factor Staining Buffer Set commercial kit instructions (00-5523-00, ThermoFisher). Then, Rat anti-Ki67-APC (652405, BioLegend) was added at 1:1000 in FACS buffer for 30 min on ice. Finally, cells were washed once more and resuspended in 200 μl of fresh FACS buffer before sample analysis by a 4L cytometer (BD LSRFortessa). Data were analyzed by FlowJo software (FlowJo v.10, Tree Star).

### Mitochondrial turnover assessment by flow cytometry

After 20 h under PDGF-BB ± SB203580 treatment, Leupeptin (L-2884, Sigma) and Ammonium chloride (A9434, Sigma) (lysosomal inhibitors) were added at 100 μM and 20 mM respectively, for 4 h at 37 °C. Then, cells were washed with 1 ml of PBS and trypsinized. Cells were centrifuged at 1250 rpm for 5 min RT the and pellet was then resuspended in 500 μl of 5% FBS DMEM with 10 nM Mitotracker-APC (M22426, ThermoFisher) and incubated for 10 min at 37 ºC. Cells were again centrifuged at 1250 rpm for 5 min RT and resuspended in 250 μl of FACS buffer (5% FBS, 5 mM EDTA in PBS). Samples were acquired with a 4L cytometer (BD LSRFortessa) and analyzed by FlowJo software (FlowJo v.10). The quantification of the mitochondrial degradation rate was carried out following previous publications^25^. Briefly, to calculate degradation rates, mean MTDR intensity values ​​obtained in the presence of lysosomal inhibitors were divided by mean intensity values ​​obtained in the absence of lysosomal inhibitors. Normalization was performed for each condition against the control condition (MT4-MMP^+/+^ without PDGF-BB or SB treatments).

### Statistics

 All statistical analysis was performed using Prism Software (GraphPad Prism 7, GraphPad software). All data are shown as mean ± s.e.m., normal distribution of the values was checked with D'Agostino-Pearson normality test and outlier values were excluded using the online GraphPad outlier test (α = 0.01). Homoscedasticity was also tested in all samples. Performed statistical analysis are detailed in each figure legend. T-tests were performed two-sided, multiple comparisons performed by one- or two-way ANOVA were followed by the Benjamini and Hochberg comparison test. Statistical significance was assigned at */#p < 0.05, **/##p < 0.01, ***/###p < 0.001 and ****/####p < 0.0001 whereas “*” denotes intra-genotype comparison and “#” denotes inter-genotype comparison.

## Supplementary Information

Supplementary Information.

## Data Availability

The datasets generated and/or analysed during the current study are available from the corresponding author on reasonable request.
