# Supplementary material for: p38 MAPK priming boosts VSMC proliferation and arteriogenesis by promoting PGC1α-dependent mitochondrial dynamics
Source: Sci Rep. 2022 Apr 8;12:5938. doi: 10.1038/s41598-022-09757-x (PMC8994030; doi:10.1038/s41598-022-09757-x)
Supplement: Supplementary file 1 — Supplementary Information. [file 41598_2022_9757_MOESM1_ESM.pdf]

Supplementary Information

p38 MAPK priming boosts VSMC proliferation and arteriogenesis by promoting PGC1α-dependent mitochondrial dynamics

Álvaro Sahún-Español, Cristina Clemente, Juan Ignacio Jiménez-Loygorri, Elena Sierra-Filardi, Leticia Herrera-Melle, Aurora Gómez-Durán, Guadalupe Sabio, María Monsalve, Patricia Boya, Alicia G. Arroyo*.

This file includes:

Supplementary Materials & Methods
Supplementary figures S1 to S13
Supplementary tables S1 to S4

Supplementary Materials & Methods:

Generation of MT4-MMPflox/flox mice

A plasmid containing Mmp17 exon 2 flanked by loxP sequences (Flox construct) (Gene Bridges) was electroporated into mouse embryonic stem cells (ESCs) from 129Sv mouse line. ESCs were then cultured in the presence of neomycin for a first screening of cells with successful plasmid recombination. Next, positively selected ESC clones were screened for correct homologous recombination by southern blot, digesting DNA with Kpn1 restriction enzyme and using specific probes at 5' (552 bp) and 3' (579 bp) flanking the homologous recombination site. Both probes were generated by amplifying genomic DNA with specific primers (Supplementary Table S1). Next, heterozygous clones were microinjected into C57BL/6 blastocysts embryos. After birth, mouse chimeras were crossed 10 times with C57BL/6 mice to obtain a pure C57BL/6 background. Finally, Neomicyn (Neo) cassette was deleted by crossing transgenic mice with flippase expressing mice (kindly provided by M. Torres' laboratory, CNIC). Specific primers were used to genotype flippase expressing mice and to corroborate Neo cassette deletion (Supplementary Table S1).

Aorta immunofluorescence

Mice were sacrificed by decapitation at P7 (7-days-old mice) and aortas were dissected, after lungs and esophagus removal, by cutting close to the dorsal spine with curved surgical scissors (14005-12, F.S.T). After cleaning from fat and blood, aortas were fixed in 4% PFA O/N at 4 ºC. Next day, aortas were washed 3 times 5 minutes in cold PBS and then incubated for 24 h at 4 ºC in 30% sucrose in PBS. Finally, aortas were embedded in OCT and frozen at

-80 ºC. For staining, 10 µm sections were cut transversally and blocked for 1 h at RT with 0.2% Triton X-100, 5% goat/donkey serum and 5% BSA in PBS. Then, primary antibodies (Supplementary Table S3) were incubated O/N at 4 ºC in 0.2% Triton X-100, 2.5% goat/donkey serum and 2.5% BSA in PBS. Next day, sections were washed at RT for 10 minutes 3 times with 0.1% Triton X-100 in PBS and secondary antibodies (Supplementary Table S3) and DAPI (1/5000) for nuclear staining were incubated for 1.5 h at RT in 0.2% Triton X-100, 2.5% goat/donkey serum and 2.5% BSA in PBS. A confocal microscope (Nikon A1R, Nikon) was used to acquire a 20x 5-z-stack tile-scan of the whole aortic section every 1.5 µm. Fiji/Image J software was used to count total number of VSMCs, defined as DAPI+ nuclei between the outer and the inner elastin lamina, and mitotic VSMCs, defined as DAPI+/Ph3+ cells.

Sequential immunostaining

When both rabbit anti-βGal and rabbit anti-Erg-647 were stained, a sequential immunofluorescence was performed. Sequential staining started by incubating rabbit anti-βGal O/N as described, followed by appropriate secondary antibody incubation next day. At this moment, and after thorough washing of the secondary antibody (10 minutes six times in 0.2% Triton X-100 in PBS), sections were re-blocked 1 h at RT in 0.2% Triton X-100, 5% rabbit serum in PBS. This avoided possible unspecific binding of the rabbit anti-Erg-647 to remnant secondary antibody. Thus, after rabbit serum re-blocking, rabbit anti-Erg-647 was incubated for 1 h at RT together with other directly marked antibodies (e.g. mouse anti-SMA-Cy3), if required. Finally, sections were washed RT for 10 minutes 3 times with 0.1% Triton X-100 in PBS, and sections were mounted as already described.

Phospho-p38 MAPK immunofluorescence

Adductor sections were washed twice 5 min in PBS and then fixed in methanol at -20ºC for 30 min. After washing in PBS twice 5 min sections were blocked and permeabilized with 0.3% Triton X-100 and 5% BSA in PBS for 1 h at RT. Next, sections were incubated for 20 min with Glycine 150 mM and washed three times with 0.1% Triton X-100 in PBS. Then, rabbit anti-p-p38 (4511, Cell Signaling) was incubated O/N 1:100 in 0.2% Triton X-100, 2.5% BSA in PBS. Next day, sections were first washed three times with 0.1% Triton X-100 in PBS and then incubated with Goat anti-rabbit 647 (A-21245, ThermoFisher) at 1:500 and DAPI at 1:5000 in 0.2% Triton X-100, 2.5% BSA in PBS. Finally, sections were washed four times 10 min in 0.1% Triton X-100 in PBS plus 10 min in

PBS and mounted in Fluoromount-G. Image acquisition and quantification was identical to the one described for VSMC proliferation assessment in adductor tissues.

Isolation of mouse aortic endothelial cells (MAECs)

After aortic digestion, MAECs and VSMCs were cultured together. After 3-4 days, MAECs will form niches and VSMCs will arrange surrounding them. Co-cultures were washed once in PBS and then were incubated 30 minutes with rat anti-ICAM-2 (553325, BD Biosciences) diluted 1:500 in PBS. Then, cells were washed twice in PBS and incubated for another 30 min at 4 ºC in culture media with Sheep anti-rat IgG magnetic beads (Dynabeads) (11035, ThermoFisher) diluted 1:250. After aspirating the beads and washing cells twice with PBS, cells were trypsinized and collected in a falcon tube inside a magnet. The supernatant (VSMCs) was removed and pellet (MAECs) was resuspended and seeded in MAECs culture media (20% FBS, 4 mM L-glutamine, 50 UI/ml penicillin, 50 µg/ml streptomycin, 25 mM HEPES and 1/50 of ECGM [C-39215, PromoCell] in DMEM). Obtained MAECs were cultured for a week changing media every three days and were used at 80% confluence without any further passage.

Tissue protein extraction

For tissue, whole aortas or pieces of adductors were collected in MagNA Lyser Green Beads tubes (03-358-941-001, Roche) being immediately frozen in liquid nitrogen. Lysis buffer was added directly to the MagnaLyser tubes, 80 µl of Ripa in the case of aortas, and 250 µl of β-Octyl (25 mM Tris-HCl pH 8, 100 mM NaCl, 1% IGEPAL, 60 mM octyl glucoside, 20 mM N-ethylmaleimide) in the case of adductor pieces, always supplemented with protease (11-836-153-001, Roche) and phosphatase (04-906-845-001, Roche) inhibitors. Tubes were placed in the MagNA Lyser (MagNA Lyser version 4.0, Roche) and 2 cycles of lysis were performed for 30 seconds at 6500 rpm. The lysate was collected and incubated for 45 minutes rotating at 4 ºC and then centrifuged at 13000 rpm, 15 minutes at 4 ºC. Supernatant was collected and quantified using Pierce BCA Protein Assay Kit (23227, ThermoFisher) and Microplate Manager software (version 5.2.1, Bio-Rad). Quantified supernatant was kept at -80ºC until used.

Tissue western blotting

A total of 60 µg of protein were reduced with 5% β-mercaptoethanol in Laemmli buffer and boiled at 100 ºC for 5 minutes. Samples were run on 8% SDS-polyacrylamide gel

for 1.5 h in running buffer (25 mM Tris-HCl pH 8.3, 192 mM glycine, 0.1% SDS) and then transferred for 1.5 h under 400 mA constant into 0.45 µm nitrocellulose membrane (1620115, Bio-Rad) in transfer buffer (25 mM Tris-HCl pH 8.3, 192 mM glycine, 20% methanol). In some experiments after blocking in 5% non-fat milk for 1 h at RT, membranes were cut prior to incubation of the fragments O/N in 2.5% non-fat milk with the corresponding primary antibodies (Supplementary Table 4). Next day, membranes were washed 3 times 5 minutes in TBST and secondary antibodies (HRP from ThermoFisher or 680 and 800 Odyssey-IRDye from LI-COR) were incubated in 2.5% non-fat Milk for 1 h RT. Afterwards, membranes are washed four times 10 minutes in TBST and signal was developed. In the case of HRP secondary antibodies, membranes were briefly incubated with Luminata Immobilon Classico (WBLUC0100, Merk) and developed with a chemiluminescence imager (LAS-4000, Life Sciences), whereas in the case of fluorescent secondary antibodies, membranes were developed using a fluorescent imager (Odyssey Infrared Imaging System, LI-COR Biosciences). In both cases, western blot images were processed and quantified with Fiji/ImageJ Software.

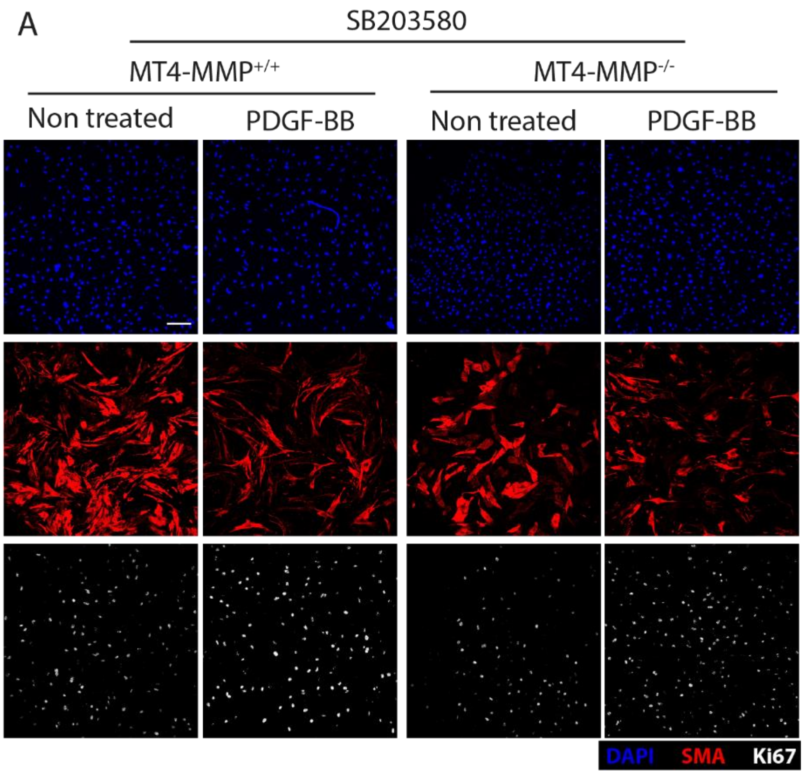

Supplementary Figure S1. Increased proliferation of aortic VSMCs lacking MT4-MMP in response to PDGF-BB. **A)** Representative confocal microscopy images showing immunostaining for SMA, Ki67 and DAPI in MT4-MMP$^{+/+}$ and MT4-MMP$^{-/-}$ aortic VSMCs left untreated or treated with PDGF-BB in presence of SB203580. Scale bar: 50 µm.

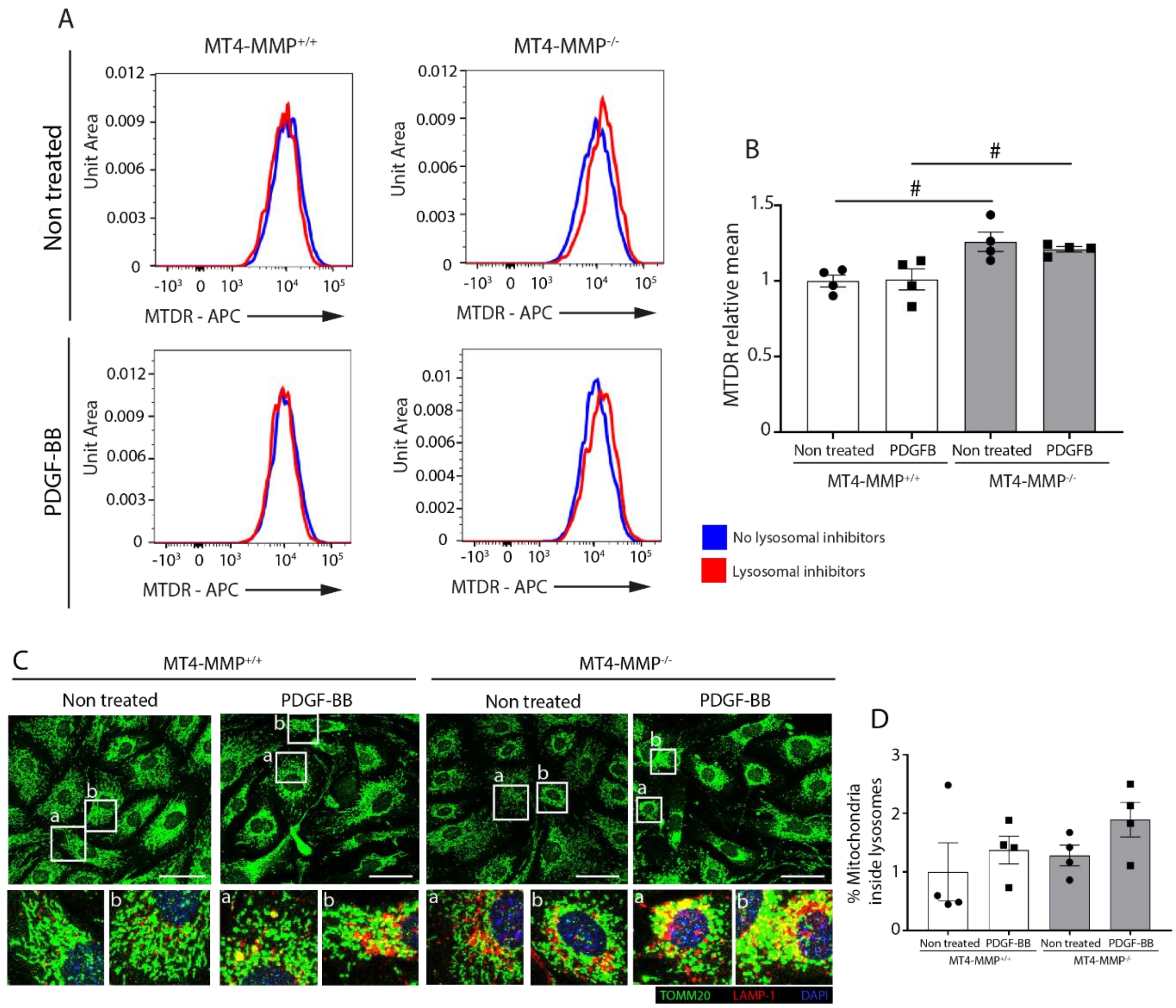

Supplementary Figure S2. Absence of MT4-MMP increases mitochondrial degradation rate in VSMCs *in vitro*. A) Representative flow cytometry histogram of MTDR signal in MT4-MMP$^{+/+}$ and MT4-MMP$^{-/-}$ aortic isolated VSMCs treated or not with PDGF-BB in presence or absence of lysosome inhibitors. **B)** Quantification of the mitochondrial degradation rate (MTDR mean intensity signal with lysosomal inhibitors divided by MTDR mean intensity signal without lysosomal inhibitors) normalized to MT4 MMP$^{+/+}$ without PDGF BB. n = 4 VSMC cultures in four independent experiments. **C)** Representative confocal microscopy images showing immunostaining for TOMM20 and LAMP1 in MT4-MMP$^{+/+}$ and MT4-MMP$^{-/-}$ aortic VSMCs left untreated or treated with PDGF. Scale bar: 50 µm. **D)** Quantification of percentage of mitochondria inside lysosomes normalized to MT4 MMP$^{+/+}$ non treated. n = 4 VSMC cultures in four independent experiments. In B and D data are means ± s.e.m. analysed by Two-way ANOVA followed by Benjamini and Hochberg post-test; $^{\#}$ $p < 0.05$.

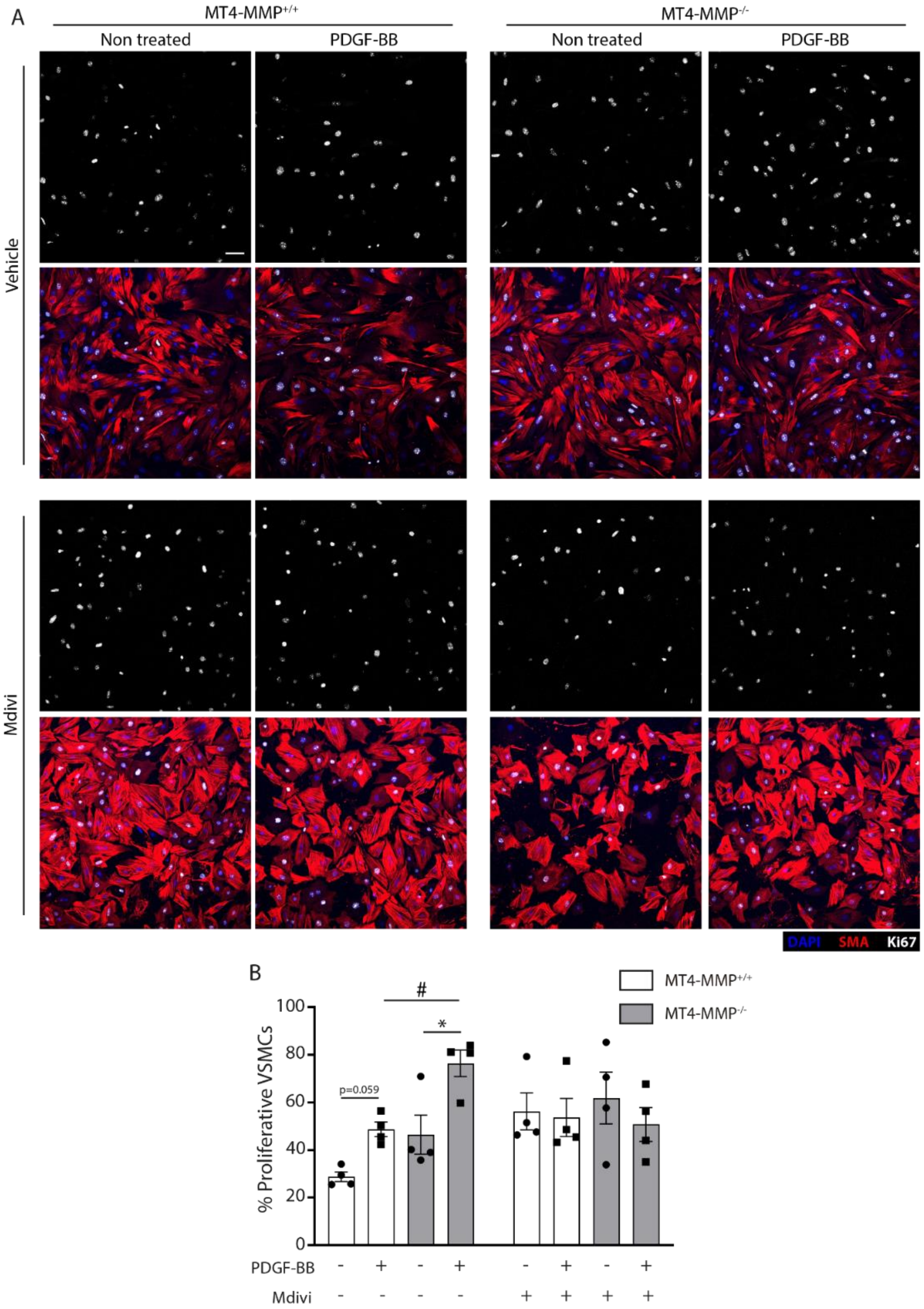

Supplementary Figure S3. Effect of inhibition of mitochondrial fission on PDGF-BB-induced VSMC proliferation. A) Representative confocal microscopy images showing immunostaining for SMA, Ki67 and DAPI in MT4-MMP$^{+/+}$ and MT4-MMP$^{-/-}$ aortic VSMCs left untreated or treated with PDGF-BB in the presence or absence of the inhibitor of mitochondrial fission mdivi-1. Scale bar: 50 µm. **B)** Quantification of the percentage of proliferative VSMCs (% Ki67^{+} in SMA^{+} cells) in MT4-MMP$^{+/+}$ and MT4-MMP$^{-/-}$ aortic isolated VSMCs treated as in **A**. n = 4 VSMC cultures in four independent experiments. Data are means ± s.e.m. analysed by Two-way ANOVA followed by Benjamini and Hochberg post-test. *, $^{\#}$ $p<0.05$.

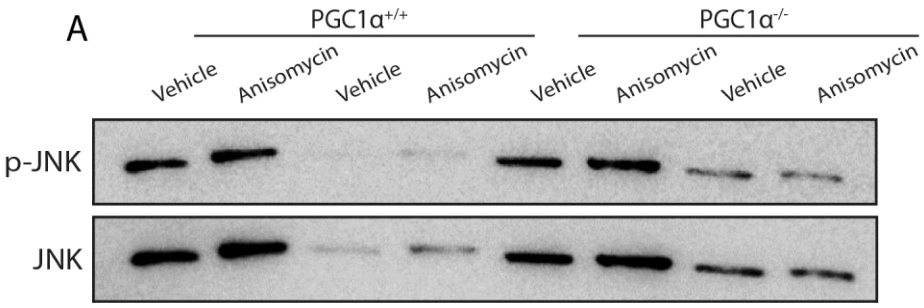

Supplementary Figure S4. Selective phosphorylation of p38 MAPK by anisomycin in cultured VSMCs from mouse aorta. A) Western blot of phospho-JNK (Thr183/Tyr185) and JNK in two independent batches of PGC1$\alpha^{+/+}$ and PGC1$\alpha^{-/-}$ VSMCs pre-treated with vehicle or anisomycin.

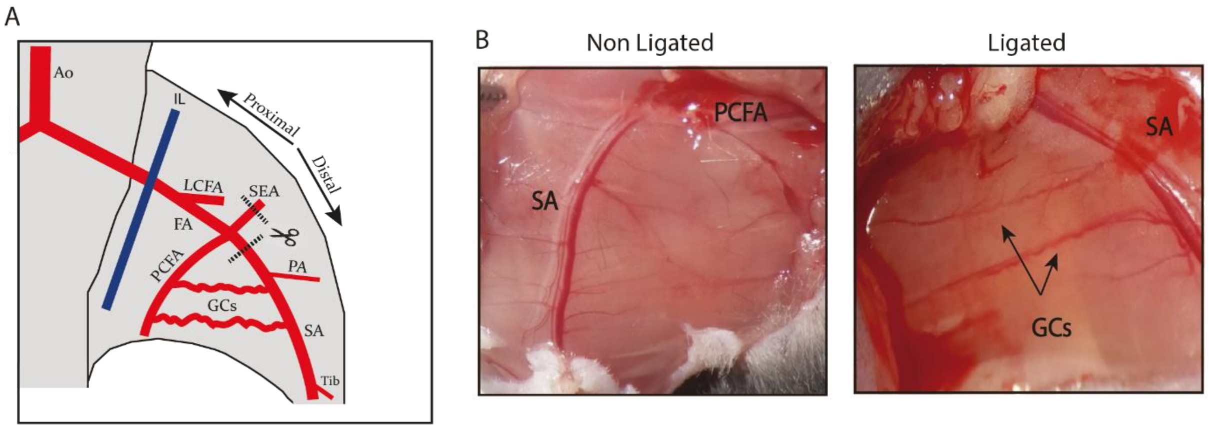

Supplementary Figure S5. HLI ischemia model. A) Simplified representation of the mouse hindlimb vascular tree. From proximal to distal: Aorta (Ao), femoral artery (FA), lateral caudal femoral artery (LCFA), proximal caudal femoral artery (PCFA), superficial epigastric artery (SEA), popliteal artery (PA), gracilis collaterals (GCs), saphenous artery (SA) and tibialis artery (Tib). **B)** Representative images showing the remodeling of the GCs after the surgery compared with a Non Ligated scenario.

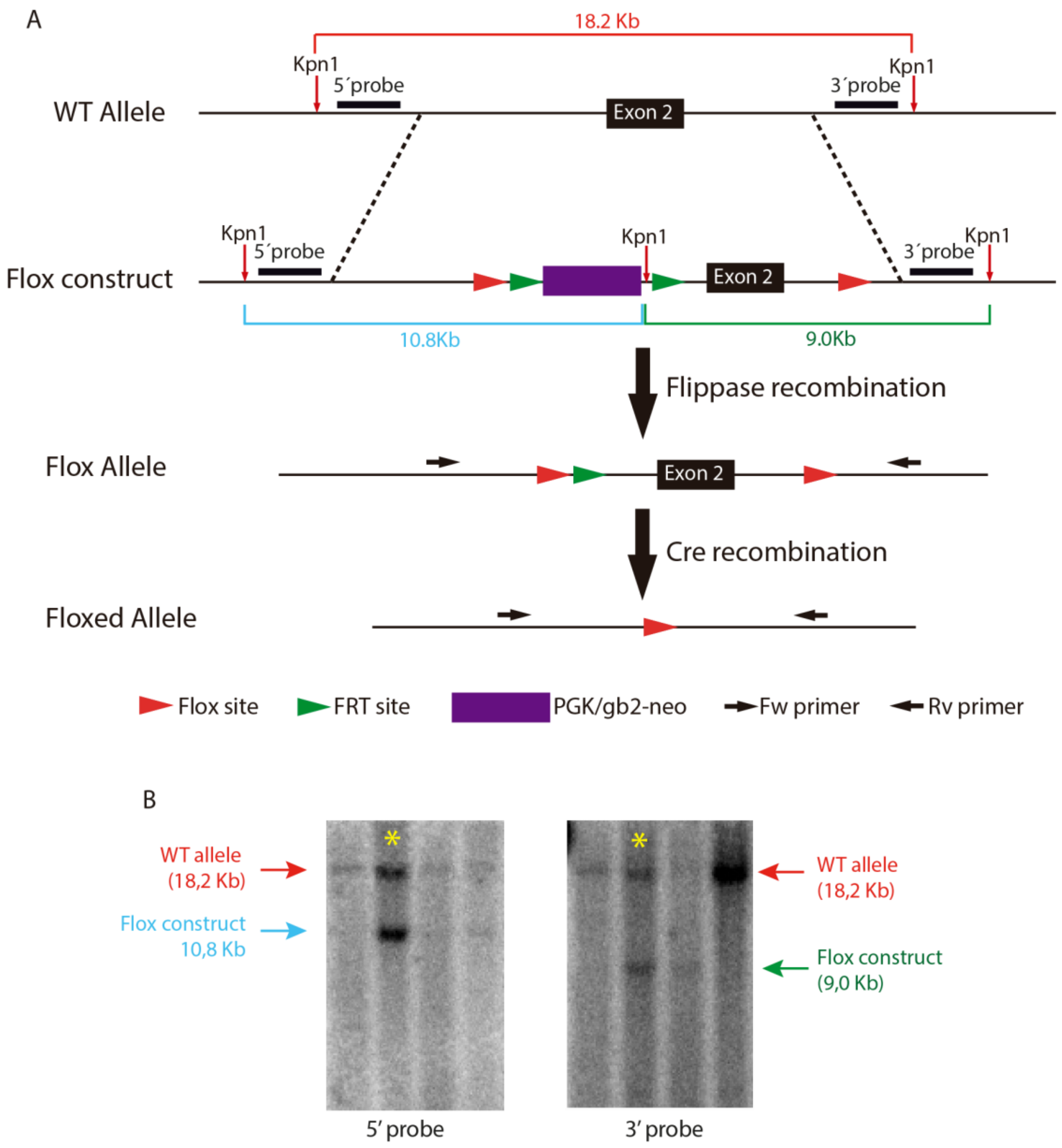

Supplementary Figure S6. Genetic generation of the MT4-MMP$^{f/f}$ mice. A) Schematic illustration of the genetic construct used for the generation of the MT4-MMP$^{f/f}$ mice.
B) Representative Southern blot of 5' and 3' probes in clonal colonies of electroporated ESCs cells. Yellow asterisks mark the clonal colony with the correct homologous recombination of the plasmid.

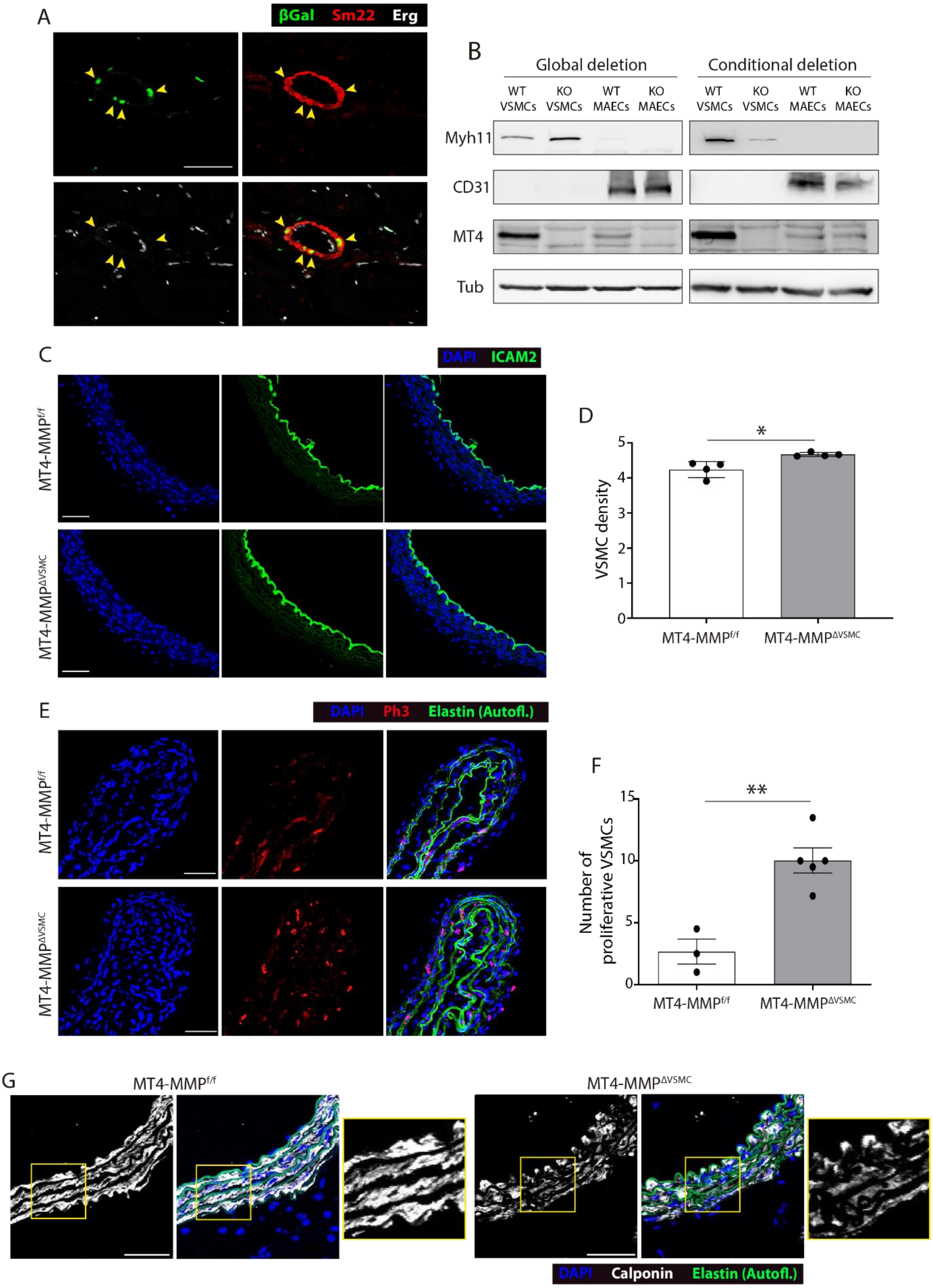

Supplementary Figure S7. Deletion of MT4-MMP in VSMCs under Sm22 promoter recapitulates the neonatal aorta phenotype of global MT4-MMP$^{-/-}$ mice.

A) Representative confocal microscopy images showing immunostaining for β-Gal (MT4-

MMP), Sm22, Erg and DAPI in MT4-MMP$^{+/-}$ mice adductors. Scale bar 50 µm. Yellow arrowheads mark Erg^{-}/SMA^{+} (defined as VSMCs) which are β Gal^{+}. **B)** Representative Western blot of MT4-MMP, Myh11, CD31 and Tubulin in lysates of cultured aortic isolated VSMCs and ECs from MT4-MMP$^{+/+}$ and MT4-MMP$^{-/-}$ (global deletion) or MT4-MMP$^{f/f}$ and MT4-MMP$^{\Delta VSMC}$ (conditional deletion). **C)** Representative confocal microscopy images showing immunofluorescence for ICAM2 and DAPI in MT4-MMP$^{f/f}$ and MT4-MMP$^{\Delta VSMC}$ aortas of neonatal (P7) mice. Scale bar: 50 µm. **D)** Quantification of VSMC nuclei per 1000 µm^2 in MT4-MMP$^{f/f}$ and MT4-MMP$^{\Delta VSMC}$ aortas. n = 4 mice of each genotype in two independent experiments. **E)** Representative confocal microscopy images showing immunostaining for phospho-Histone H3, DAPI and auto-fluorescent elastin in MT4-MMP$^{f/f}$ and MT4-MMP$^{\Delta VSMC}$ aortas of neonatal (P7) mice. Scale bar: 50 µm. **F)** Quantification of mitotic (Ph3^{+}) VSMCs in MT4-MMP$^{f/f}$ and MT4-MMP$^{\Delta VSMC}$ aortas. n = 3 MT4-MMP$^{f/f}$ mice and 5 MT4-MMP$^{\Delta VSMC}$ mice in one experiment. **G)** Representative confocal microscopy images showing immunostaining for Calponin, DAPI and auto-fluorescent elastin in MT4-MMP$^{f/f}$ and MT4-MMP$^{\Delta VSMC}$ aortas of adult mice. Scale bar: 50 µm. In D and F data are means ± s.e.m. analyzed by unpaired t test. * $p<0.05$, ** $p<0.01$.

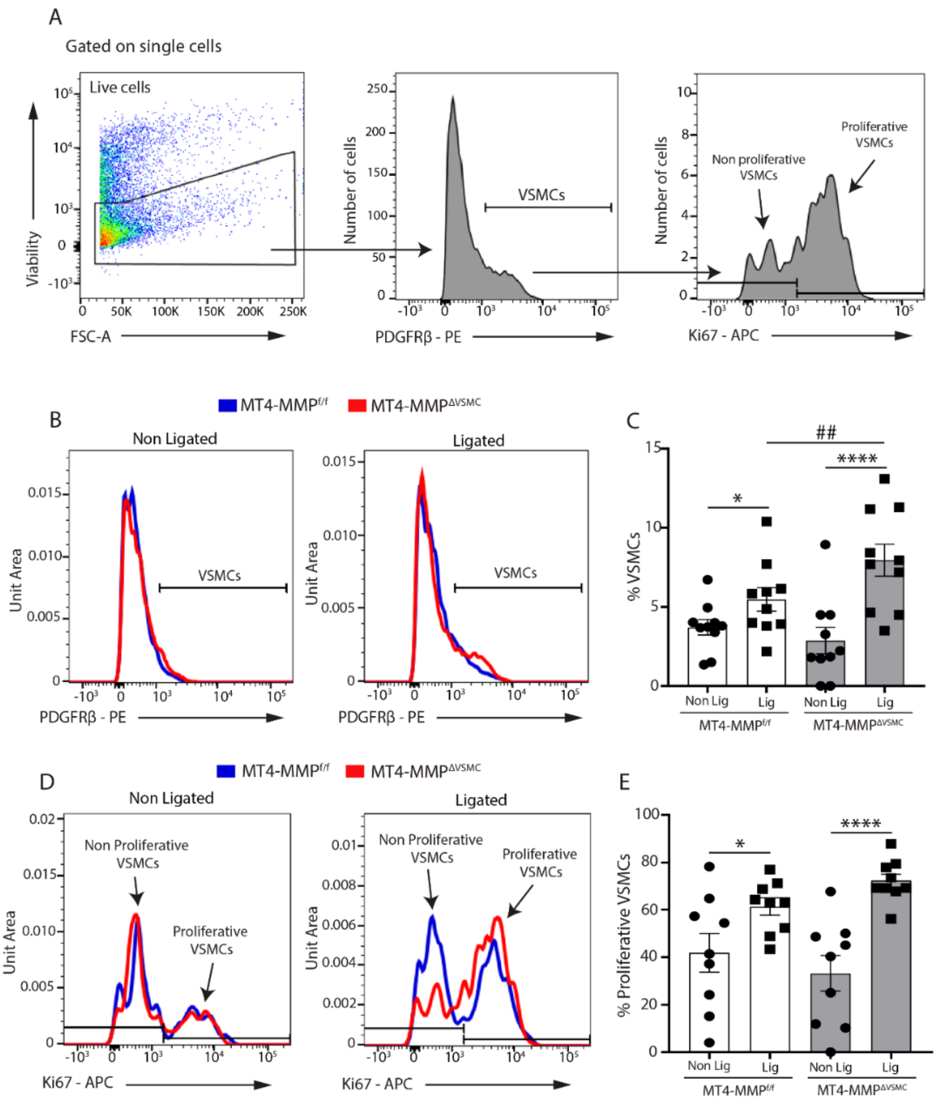

Supplementary Figure S8. Specific MT4-MMP deletion in VSMCs increases PDGFRβ^{+} VSMC numbers in superficial adductor muscle after femoral artery ligation. A) Gating strategy used to assess VSMC presence and its specific proliferation in superficial adductor muscles 7 days after surgery. **B)** Representative flow cytometry histogram plots of PDGFRβ staining in Non Ligated and Ligated superficial adductor muscles of MT4-MMP$^{f/f}$ and MT4-MMP$^{\Delta VSMCs}$ mice. **C)** Quantification of the percentage of VSMCs (PDGFRβ^{+}). n = 10 mice per genotype in 5 independent experiments. **D)** Representative flow cytometry histogram plots of Ki67 staining in Non-Ligated and Ligated superficial adductor muscles of MT4-MMP$^{f/f}$ and MT4-MMP$^{\Delta VSMCs}$ mice. **E)** Quantification of the percentage of proliferative VSMCs (PDGFRβ^{+}/Ki67^{+}). n = 9 mice per genotype in 5 independent experiments. Scale bar: 50 µm. In C and E data are means ± s.e.m. analysed by Two-way ANOVA followed by Benjamini and Hochberg post-test; * $p<0.05$, $^{\#\#}$ $p<0.01$, **** $p<0.0001$.

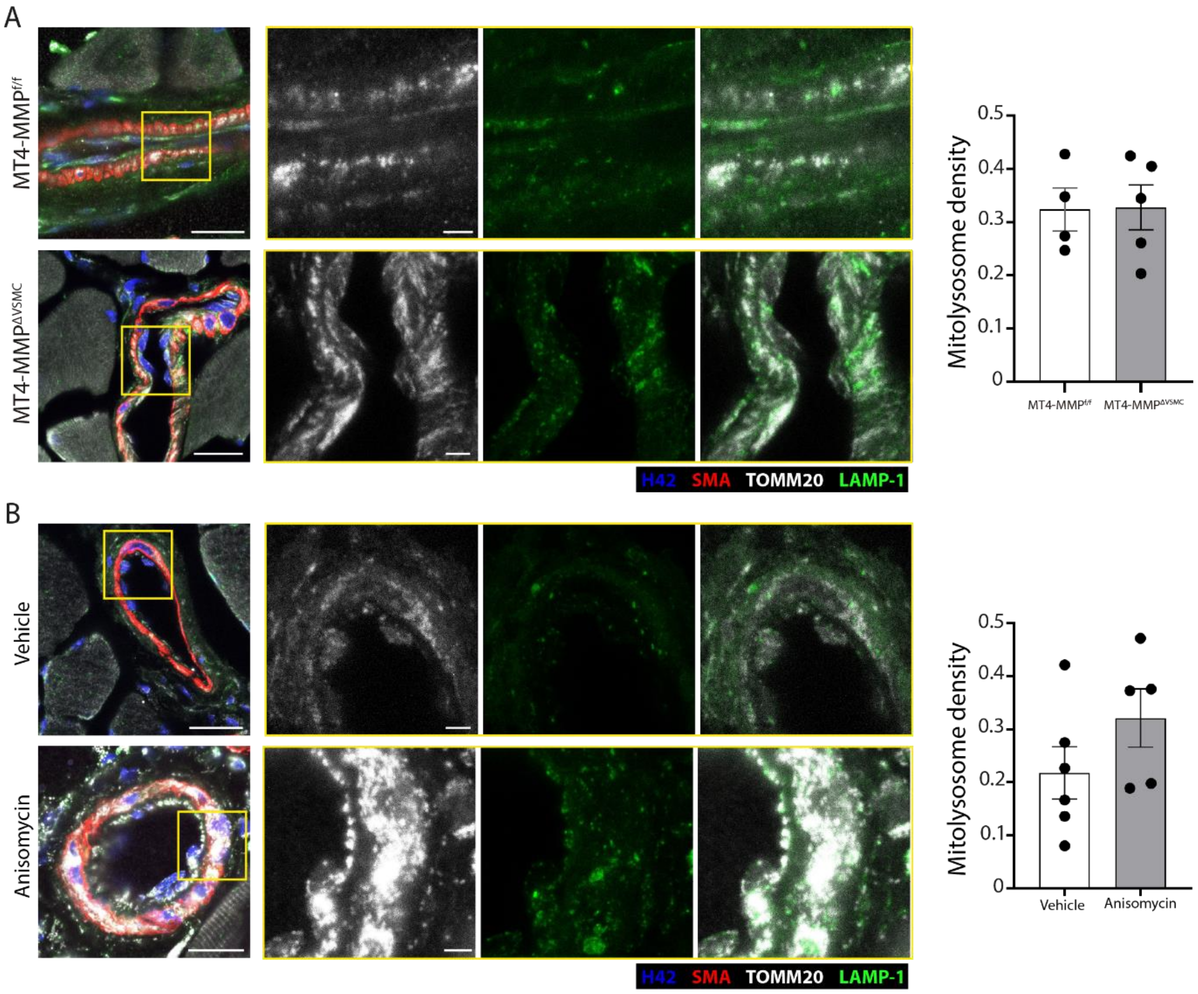

Supplementary Figure S9. Abundance of mito-lysosomes in VSMCs from remodeled arterioles after femoral artery ligation. Representative confocal microscopy images showing immunostaining for SMA, TOMM20, LAMP1, and DAPI (left) and mitolysosome quantification (right) in VSMCs within the remodeled arterioles of ligated adductor muscles from MT4-MMP$^{f/f}$ or MT4-MMP$^{\Delta VSMC}$ mice (**A**) and from vehicle- or anisomycin-treated mice (**B**). An enlarged image is also included to better show the signal from mitochondria (TOMM20^{+}) and lysosomes (LAMP1^{+}) in VSMCs (SMA^{+}) in arterioles. Scale bar in zoomed-out visualization: 25 µm. Scale bar in zoomed-in visualization: 5 µm. Data are means ± s.e.m. analysed by unpaired t-test.

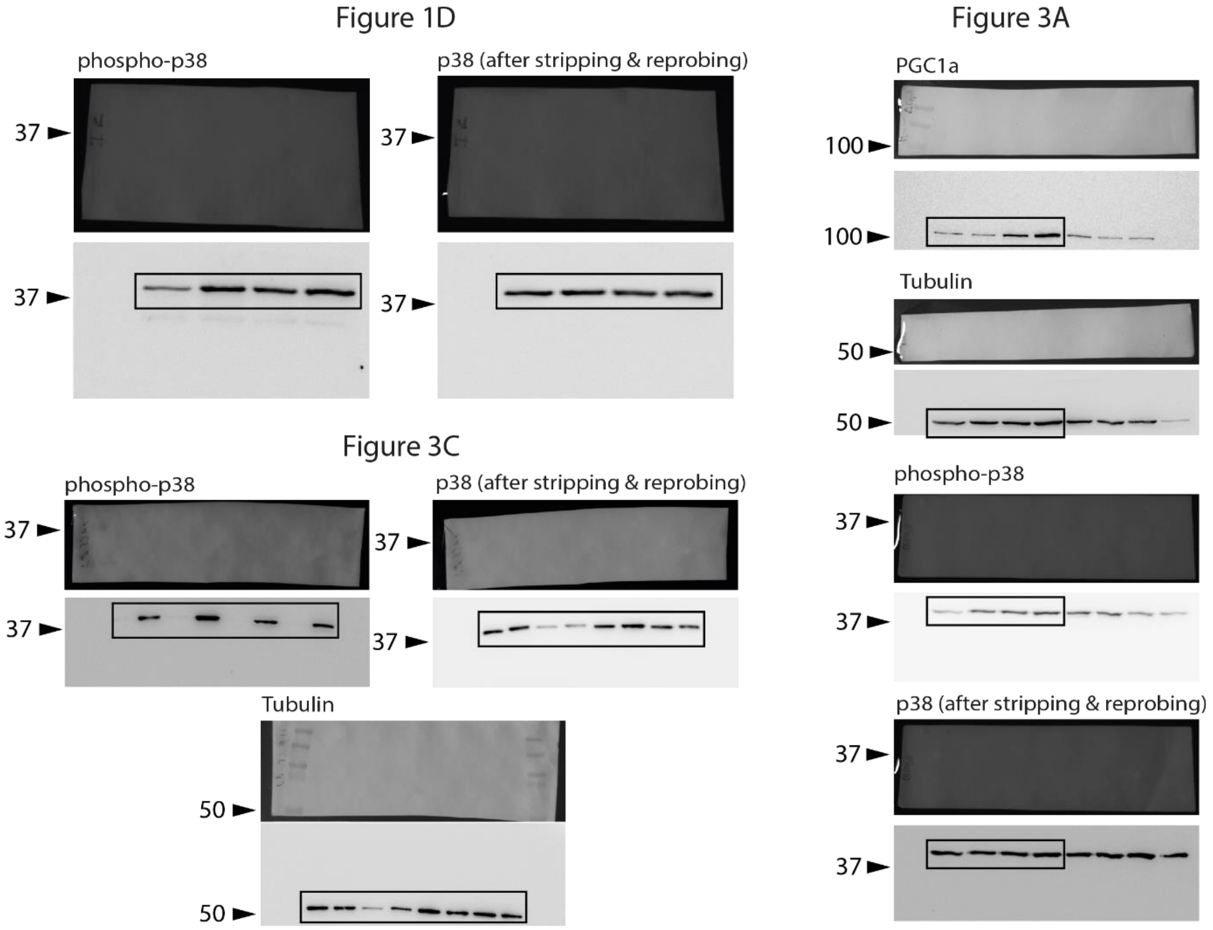

Supplementary Figure S10. Uncropped western blots used for the main figures.

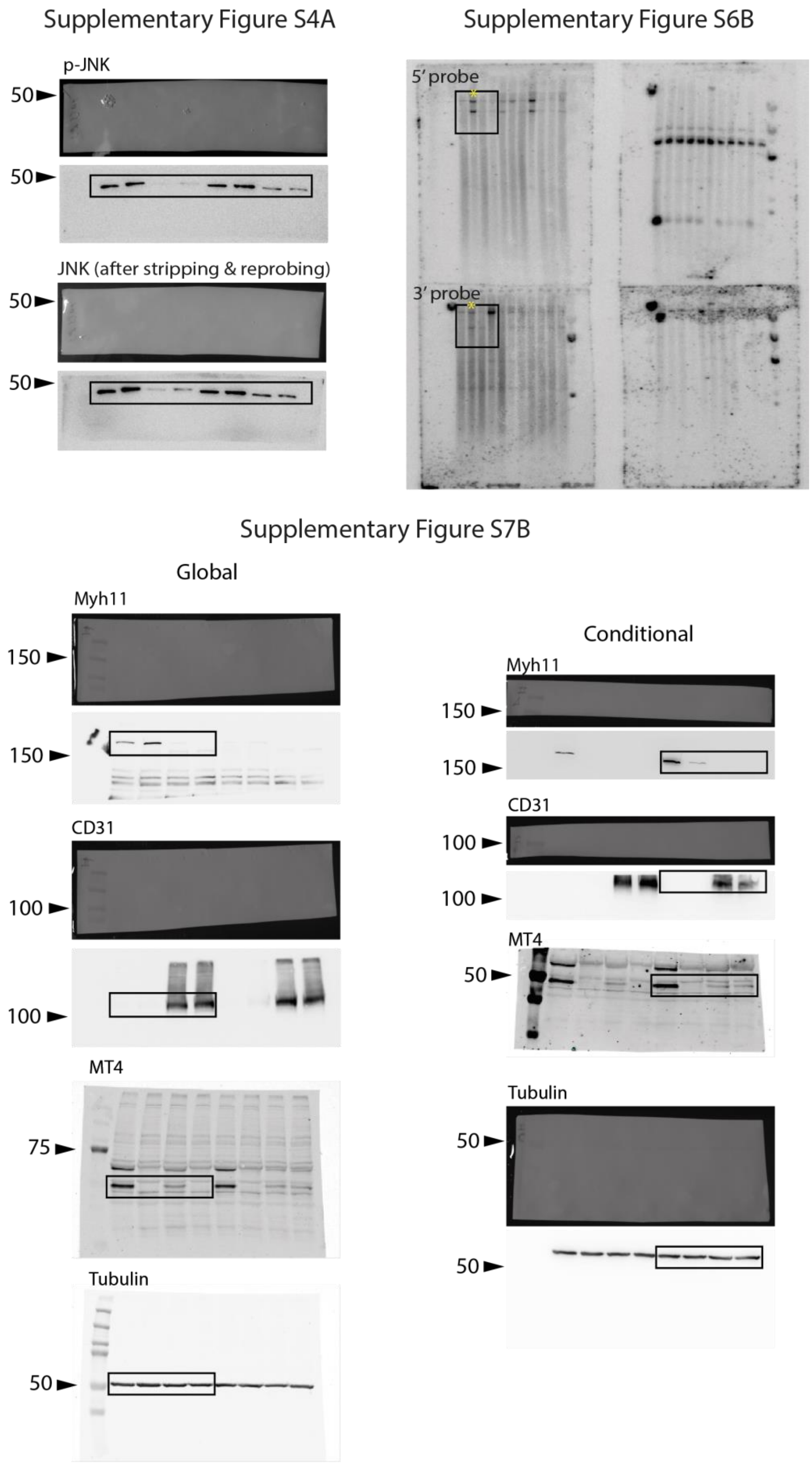

Supplementary Figure S11. Uncropped western blots used for the supplementary figures.

Fig 1D: 4 independent experiments

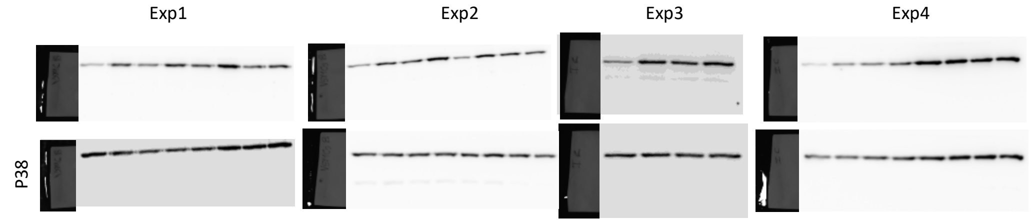

Selected representative: Exp3
For graph Fig 1E: Quantified only 4 first wells (0% FBS)

Sample order (if only 4, the first 4)	0% FBS				1% FBS			
	WT N	WT P	KO N	KO P	WT N	WT P	KO N	KO P

Fig 3A: 6 independent experiments

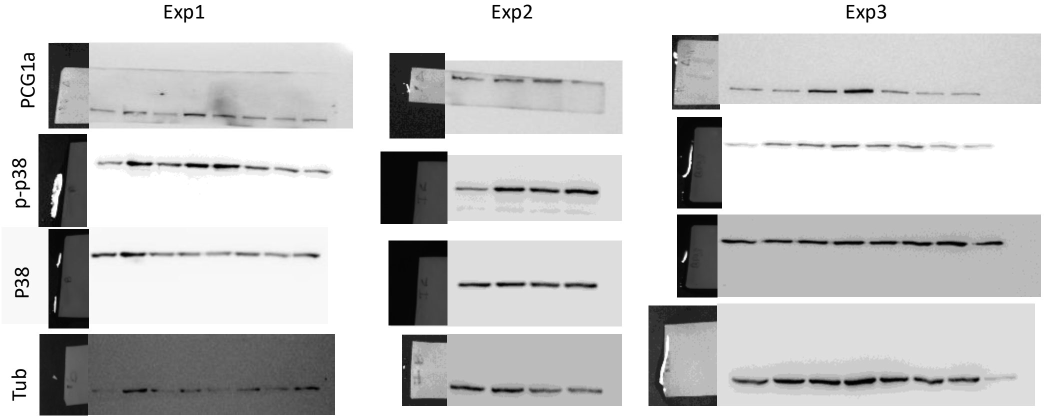

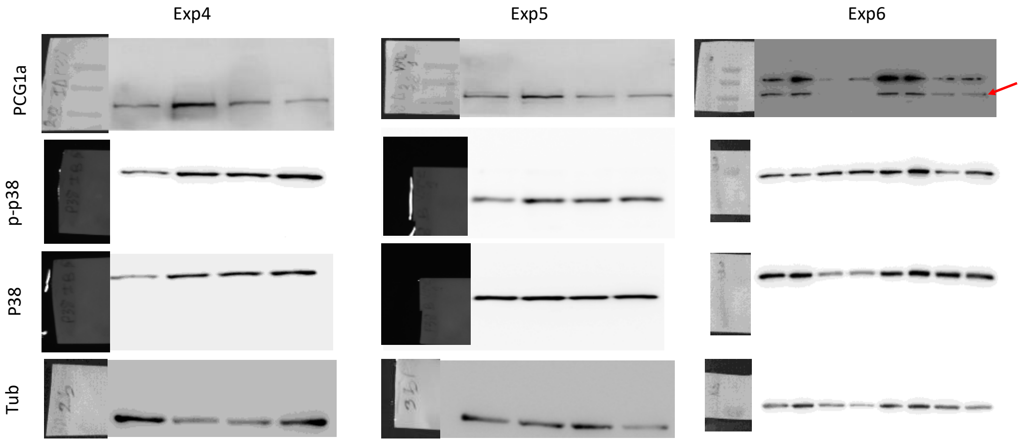

Selected representative: Exp3
For graphs Fig 3B: Quantified only 4 first wells (no SB) and shown by correlation

Sample order (if only 4, the first 4)	WT N -	WT P -	KO N -	KO P -	WT N SB	WT P SB	KO N SB	KO P SB

Fig 3C: 1 experiment, two replicates per genotype

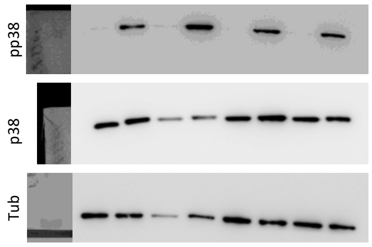

Selected representative: only image
No graph associated

Sample order	WT1		WT2		KO1		KO2	
	basal	2h	basal	2h	basal	2h	basal	2h

Supplementary Figure S12. Replicates of western blots used for the main figures.

Supplementary Fig S4: 1 experiment, two replicates per genotype

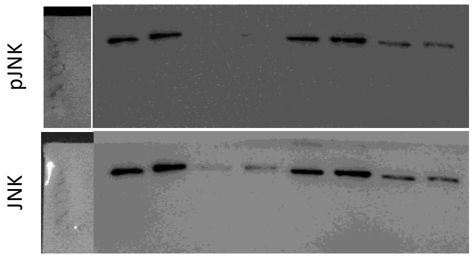

Selected representative: only image
No graph associated

Sample order	WT1		WT2		KO1		KO2	
	basal	2h	basal	2h	basal	2h	basal	2h

Supplementary Fig S6: 1 experiment

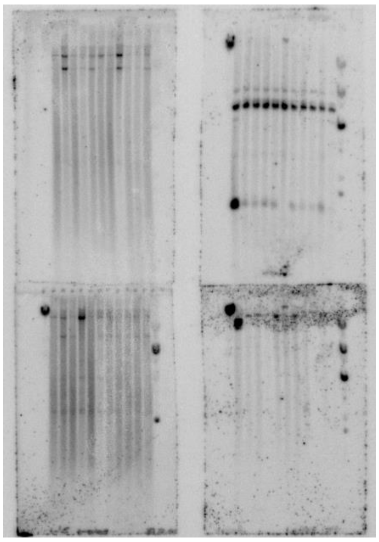

Sample order (from left to right):
1-λH
2-clon 68
3-clon 99
4-clon 101
5-clon 121
6-clon 140
7- clon 203
8-clon 204
9-clon 209
10-clon 227
11- clon 256
12- clon 272
13-λB

Selected representative: only image
No graph associated

Supplementary Fig S7B: 4 independent experiments

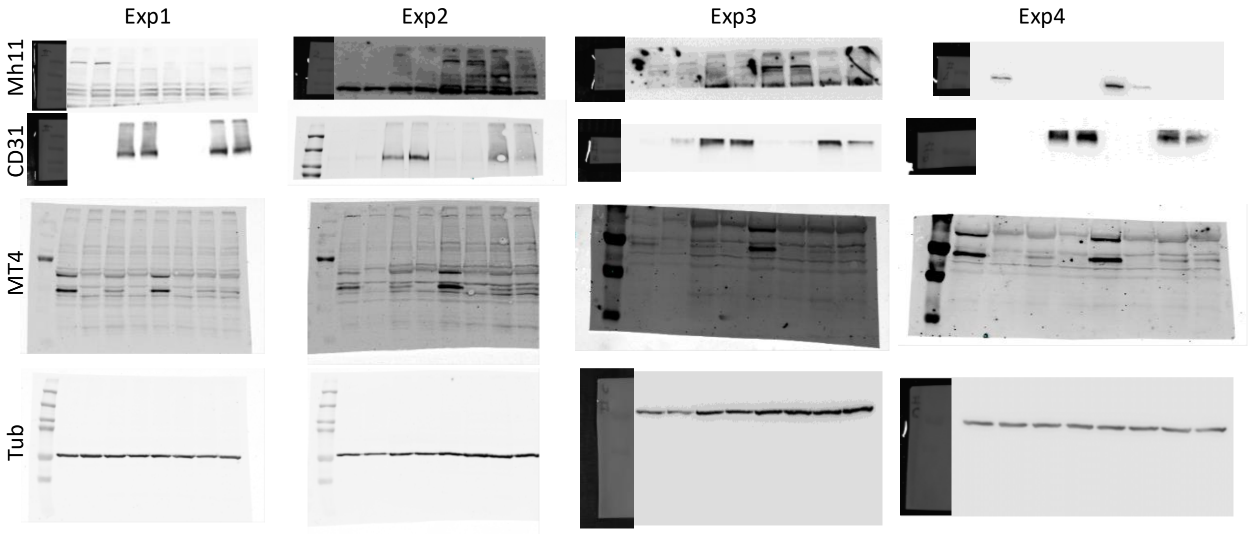

Selected representative: for global (exp 1) and for conditional (Exp4) [they are represented independently in supplementary Fig 7B]. No graph associated

Sample order	Global				Conditional			
	VSMCs WT	VSMCs KO	ECs WT	ECs KO	VSMCs WT	VSMCs KO	ECs WT	ECs KO

Supplementary Figure S13. Replicates of western blots used for the supplementary figures.

Supplementary Table S1. Sequence of the genotyping primers.

Mouse line	Primer name	Primer sequence (5’ – 3’)
MT4-MMP$^{lacZ/lacZ}$	Forward WT	TCAGACACAGCCAGATCAGG
	Forward KO	AATATGCGAAGTGGACCTGG
	Reverse (common)	AGCAACACGGCATCCACTAC
MT4-MMP$^{flox/flox}$	Forward	CCTAATGTACATAGCCAGCCAAG
	Reverse	AAGGCCAGGTGTGTTCAATC
MT4-MMP$^{\Delta VSMC}$	Reverse (Cre specific)	CGCATAACCAGTGAAACAGCATTGC
	Forward (Sm22 specific)	CAGACACCGAAGCTACTCTCCTTCC
PGC1α - KO	Forward	TCCAGTAGGCAGAGATTTATGAC
	Reverse	CCAACTGTCTATAATTCCAGTTC
PGC1α - WT	Forward	CTTCCATGTGTCACTGTAGTC
	Reverse	GGATGATAGGTATGCGTTAC
MT4-MMP 5’ probe	Forward	GTCAGCCCCTCATATTTCATTTAC
	Reverse	GGTCTGGCTCAATCTCTGTTTAAT
MT4-MMP 3’ probe	Forward	CTGGTGTTTCAGGTTTAGAGGAGT
	Reverse	AAGACAATCTTGTGGTCACTGGTA
Flippase	Forward	CTAATGTTGTGGGAAATTGGAGC
	Reverse	CTCGAGGATAACTTGTTTATTGC
Neo cassette	Forward	AGGATCTCCTGTCATCTCACCTTGCTCCTG
	Reverse	AAGAACTCGTCAAGAAGGCGATAGAAGGCG

Supplementary Table S2. Primary and secondary antibodies used in VSMC immunofluorescence.

Antibody	Concentration	Provider	Ref.
Mouse anti-SMA-Cy3	1:400	Sigma-Aldrich	C6198
Rabbit anti-Ki67	1:100	Abcam	ab16667
Rat anti-LAMP1	1:100	DSHB	1D4B
Rabbit anti-TOMM20	1:100	Santa Cruz	sc-11415
Goat anti-Rabbit Alexa 488	1:200	Molecular Probes	A-11008
Goat anti-Rat Alexa 568	1:200	Molecular Probes	A-11077
Goat anti-Rabbit Alexa 647	1:500	ThermoFisher	A-21245

Supplementary Table S3. Primary and secondary antibodies used in tissue immunofluorescence.

Antibody	Concentration	Provider	Ref.
Rabbit anti-βGal	1:100	Abcam	ab4761
Rat anti-ICAM2	1:200	BD Biosciences	553325
Goat anti-Sm22	1:100	Abcam	ab10135
Rabbit anti-Erg	1:100	Abcam	ab92513
Rabbit anti-pH3	1:100	Merck/Millipore	06-570
Mouse anti-SMA-Cy3	1:400	Sigma	C6198
Rabbit anti-Erg-647	1:100	Abcam	ab196149
Rabbit anti-phospho-p38	1:100	Cell Signalling	4511
Rabbit anti-Calponin	1:100	Abcam	ab46794
Rabbit anti-PGC1α	1:100	Santa Cruz	sc-13067
Rat anti-LAMP1	1:100	DSHB	1D4B
Rabbit anti-TOMM20	1:100	Santa Cruz	sc-11415
Goat anti-Rabbit Alexa 546	1:500	ThermoFisher	A-11035
Goat anti-Rat Alexa 488	1:500	ThermoFisher	A-11006
Donkey anti-Goat Alexa 568	1:500	ThermoFisher	A-11057
Goat anti-Rabbit Alexa 488	1:500	ThermoFisher	A-11034
Goat anti-Rabbit Alexa 647	1:500	ThermoFisher	A-21245

Supplementary Table S4. Primary antibodies used in WB.

Antibody	Concentration	Provider	Ref.
Rabbit anti-MT4-MMP	1:1000	Abcam	ab51075
Rabbit anti-CD31	1:1000	Abcam	ab28364
Mouse anti-Myh11	1:1000	Abcam	ab683
Rabbit anti-p38	1:1000	Santa Cruz	SC535
Rabbit anti-phospho-p38	1:1000	Cell Signaling	9211
Rabbit anti-JNK	1:1000	Cell Signaling	9252
Rabbit anti-phospho-JNK	1:1000	Cell Signaling	9251
Mouse anti-PGC1α	1:1000	Sigma	ST1202
